# Vulnerability of cocoa-based agroforestry systems to climate change in West Africa

**DOI:** 10.1038/s41598-023-37180-3

**Published:** 2023-06-20

**Authors:** Antonio Jesús Ariza-Salamanca, Rafael M. Navarro-Cerrillo, José L. Quero-Pérez, Belinda Gallardo-Armas, Jayne Crozier, Clare Stirling, Kauê de Sousa, Pablo González-Moreno

**Affiliations:** 1 Department of Forest Engineering, Laboratory of Dendrochronology, Silviculture and Global Change, DendrodatLab-ERSAF, University of Cordoba, Campus de Rabanales, Crta. IV, Km. 396, 14071 Córdoba, Spain; 2 Instituto Pirenaico de Ecología - CSIC ES, Zaragoza, Spain; 3 CABI, Bakeham Lane, Egham, Surrey, TW20 9TY UK; 4 Mondelez UK R&D Limited, Bournville, B30 2LU UK; 5 Department of Agricultural Sciences, Inland Norway University of Applied Sciences, 2322 Hamar, Norway; 6 Digital Inclusion, Bioversity International, Montpellier, France

**Keywords:** Agroecology, Biodiversity, Climate-change ecology, Ecological modelling, Climate change

## Abstract

Previous research indicates that some important cocoa cultivated areas in West Africa will become unsuitable for growing cocoa in the next decades. However, it is not clear if this change will be mirrored by the shade tree species that could be used in cocoa-based agroforestry systems (C-AFS). We characterized current and future patterns of habitat suitability for 38 tree species (including cocoa), using a consensus method for species distribution modelling considering for the first time climatic and soil variables. The models projected an increase of up to 6% of the potential suitable area for cocoa by 2060 compared to its current suitable area in West Africa. Furthermore, the suitable area was highly reduced (14.5%) once considering only available land-use not contributing to deforestation. Regarding shade trees, 50% of the 37 shade tree species modelled will experience a decrease in geographic rate extent by 2040 in West Africa, and 60% by 2060. Hotspots of shade tree species richness overlap the current core cocoa production areas in Ghana and Côte d’Ivoire, suggesting a potential mismatch for the outer areas in West Africa. Our results highlight the importance of transforming cocoa-based agroforestry systems by changing shade tree species composition to adapt this production systems for future climate conditions.

## Introduction

Nearly 70% of the world's cocoa production is originated in West Africa. At present, Côte d’Ivoire and Ghana are the largest producers followed by Nigeria and Cameroon. Just in Côte d’Ivoire, cocoa farms employ more than 18% of the inhabitants of the country^1^. Considering that West Africa has a drier climate than other major global cocoa origins and that this is considered a yield-limiting factor^2^, the prospect of lower rainfall induced by climate change threatens the livelihoods of millions of persons in this region.

Cocoa (*Theobroma cacao* L.) is grown on a wide range of soils and the suitability of soils varies with the climate. For example, where rainfall is well distributed the moisture-holding capacity of the soil is less important than in countries with a dry season. Therefore, it is not possible define precise soil requirements^2^. Even more relevant than total rainfall, it is the seasonality and duration of the dry seasons, which could be a major limit to cocoa suitability. In fact, more than three months with less than 100 mm rainfall seems to be a critical limit for cocoa yield and growth^2^. The rise in temperature and increasing frequency of severe droughts predicted for the near future^3^ will make cocoa plantations more vulnerable, ultimately affecting their vigour, yield and long-term persistence. As a result, some important cocoa cultivation areas in West Africa in the twentieth century might become unsuitable for growing cocoa by the near future^4–6^.

The inclusion of shade trees in cocoa plantations—agroforestry—is often proposed as a climate-smart strategy for allowing agriculture to both adapt to and mitigate climate change^7^. For instance, Niether et al.^8^ found that agroforestry systems can buffer extreme climate events in comparison to full-sun monocultures. Overall, cocoa-based agroforestry systems (hereafter C-AFS) with an optimal shade management provide several benefits to cocoa production in relation to microenvironment amelioration such as reduction of air and soil temperature extremes (heat at lower elevations and cold at higher elevations), reduction of winds speeds, buffering of humidity and soil moisture availability, and improving or maintaining the soil fertility including erosion reduction^9,10^.

Furthermore, the implementation of C-AFS might result in a diversification of incomes for the farmer and ecological improvement as the shade species might provide additional benefits such as timber, fruit or nitrogen fixation^11,12^. However, climate change can also affect the potential distribution of these shade tree species and may reduce the availability of agroforestry systems as a viable approach for climate adaptation^13^.

In this context, the modelling approach based on species distribution models (SDMs) has been widely used to predict current and future potential species distribution under a range of climate scenarios^14,15^. However, the use of SDMs should be revisited, especially in relation to the relevance of other key environmental factors besides climate^16^. For instance, the ecological preferences of plant species for particular soil properties are known to influence plant distributions^17^. The chemical properties of the soil determine nutrient availability and soil toxicity for plants^18^. On the other hand, soil physical properties such as texture or stone content have an impact on plants by promoting or limiting growth^19^. Despite the relevance of soil characteristics particularly for species of agronomy interest, few studies have considered these factors into species distribution modelling with most of the effort concentrated on bioclimatic variables alone^4–6,13^.

In this study, we used a consensus method for species distribution modelling including bioclimatic and edaphic variables, to assess the vulnerability of cocoa potential distribution under climate change as well as the potential impacts of climate change on the habitat suitability for 37 species commonly used in C-AFS across West Africa. Considering a general increase in aridity and associated risks^3^, we hypothesized that overall habitat suitability will be reduced for all species considered in this study with higher impact for genuine tropical species. Specifically, we aimed: (i) to assess the relative importance of bioclimatic and edaphic factors regarding their habitat suitability in West Africa; (ii) to predict current and future suitable habitat distribution of cocoa and associated shade trees under projected future climate changes (2040 and 2060) and (iii) to identify current and future priority areas for C-AFS based on cocoa habitat suitability and hotspots of shade species typology (e.g. timber, leguminous and fruit). The results of this study can be directly used in agroforestry systems as inputs for decision support systems, particularly in the selection of shade tree species, plantations design and management planning.

## Results

### Performance of the SDM and environmental predictors

**Figure 1:**
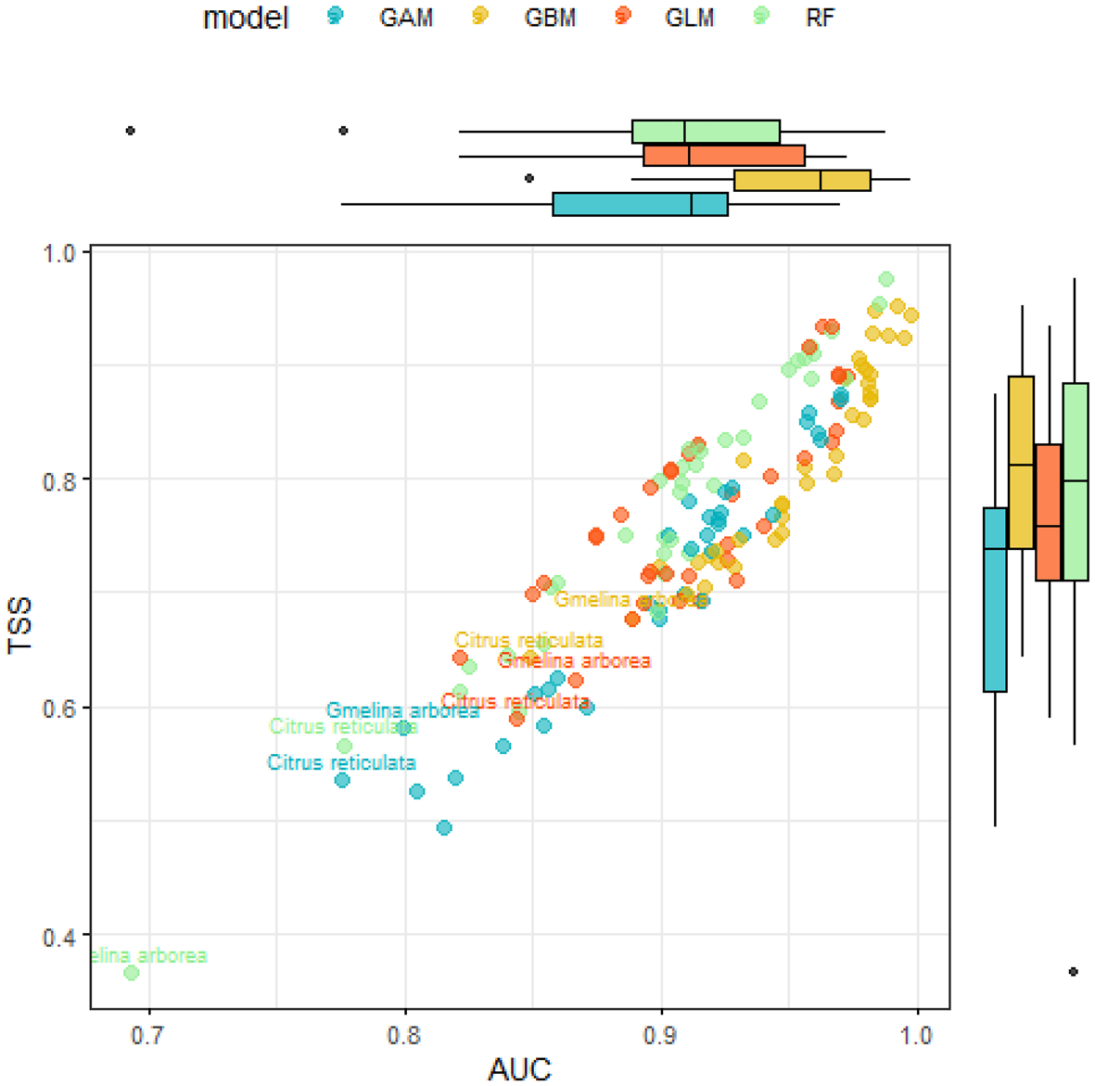
TSS and AUC (unitless) for cocoa and shade tree species according to algorithm included in the consensus species distribution model approach. Species names are shown when AUC is less than 0.8 in at least one algorithm. Models implemented: Generalized Boosted Regression Models (GBM), Generalize Additive Models (GAM), Generalized Linear Models (GLM) and Random Forest (RF).

The prediction accuracy of the selected SDMs for cocoa and shade trees showed values between 0.69 and 0.99. Model fit was generally good for all models and species (Fig. 1). Among the models, GBM (Generalized Boosted Regression Models) yielded results with the highest mean AUC values for each species. For the AUC comparison, GBM also was the most consistent in its performance, as indicated by small standard deviation (0.034). The final ensemble model that was built using all the data had an AUC range from 0.84 to 0.99 for the different species, while TSS value ranged from 0.66 to 0.96 for the ensemble models.

**Figure 2:**
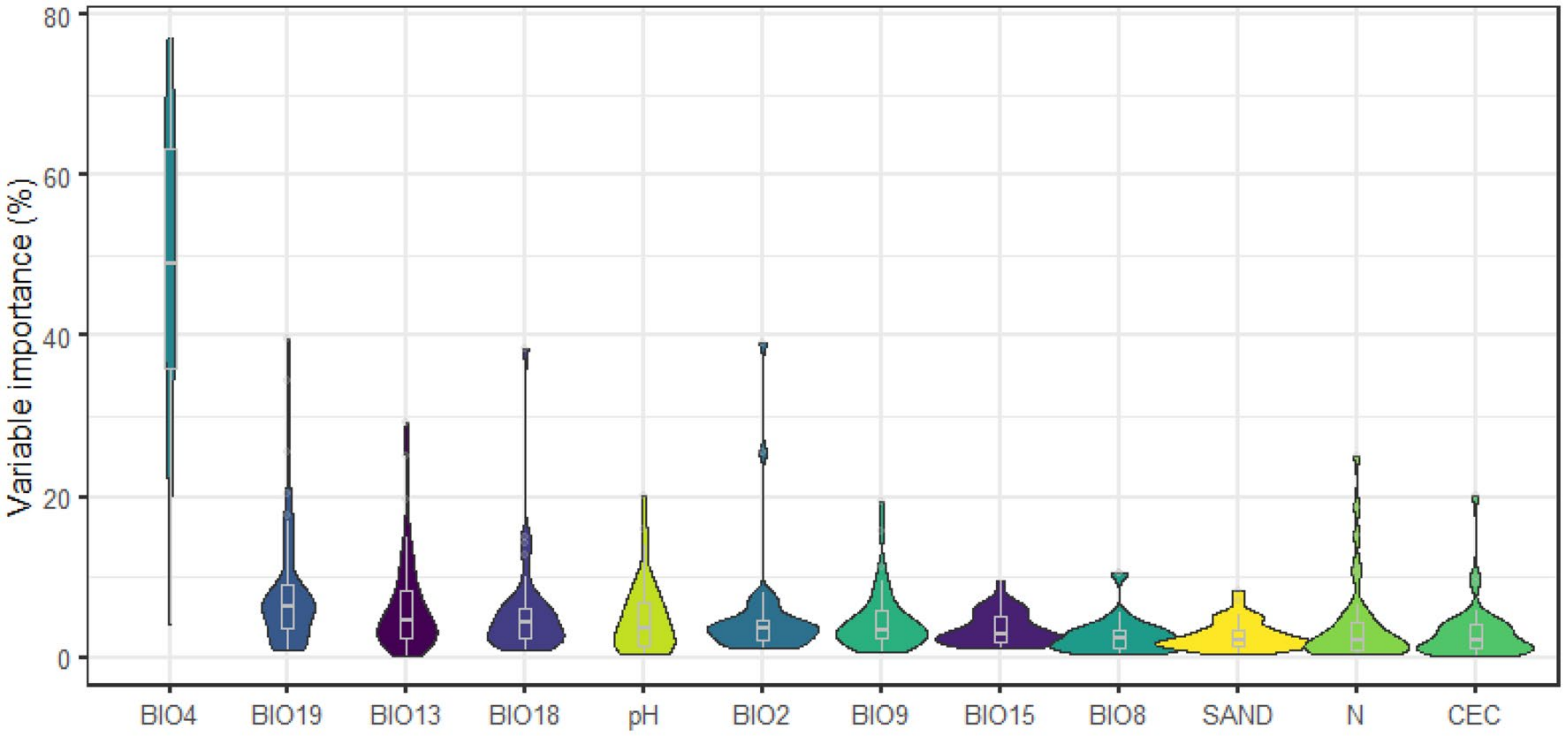
Variable importance (%) in species distribution model of cocoa and shade tree species considering the GBM algorithm. Variables are ordered according to the median of variable importance for the 38 species modelled. For variable information see Table 1.

Across all 38 tree species (cocoa and 37 shade trees), the impact of climatic variables was much greater than that of edaphic variables (Fig. 2). Specifically, the sum of the contribution rate of the bioclimatic variables reached 85% and that of the soil factors only 15%. The highest scores of the environmental variables were related to temperature seasonality (Fig. 2), except for *Acacia mangium* Willd., *Citrus grandis* (L.) Osbeck, *Citrus reticulata* Blanco and *Gmelina arborea* Roxb. ex Sm. Particularly for *Theobroma cacao* L., the sum of the contribution rate of temperature seasonality and the precipitation of warmest quarter reached 65%.

For species such as *Albizia guachapele* (Kunth) Dugand, *Citrus grandis* (L.) Osbeck, *Citrus reticulata* Blanco, *Gliricidia sepium* (Jacq.) Kunth, *Pachylobus edulis* G.Don, and *Persea americana* Mill., the contribution rate of the edaphic predictors was ~ 30%, while for other species (*Cola acuminata* (P.Beauverd) Schott & Endl., *Cola nitida* (Vent.) Schott & Endl., *Irvingia gabonensis* (Aubry-Lecomte ex O'Rorke) Baill. or *Triplochiton scleroxylon* K.Schum.), the contribution rate was < 4%. The distribution of native species was more constrained by climate than exotic species (see supplementary material Figs. S1), with the exception of *Pachylobus edulis* G.Don, whose distribution is highly conditioned by soil pH (Table S4). In contrast, we did not find any significant difference in relative importance between climatic and edaphic across the main use of the species (see supplementary material Figs. S2, Table S4). Within the edaphic variables, pH was also relatively strong predictor of species occurrence while sand and nitrogen content, and cation exchange capacity (CEC) were the least important (Fig. 2). However, their importance varied among species. Nitrogen soil content and CEC were the strongest predictors of occurrence for *Theobroma cacao* L., followed by pH and sand content.

### Changes in habitat suitability

**Figure 3:**
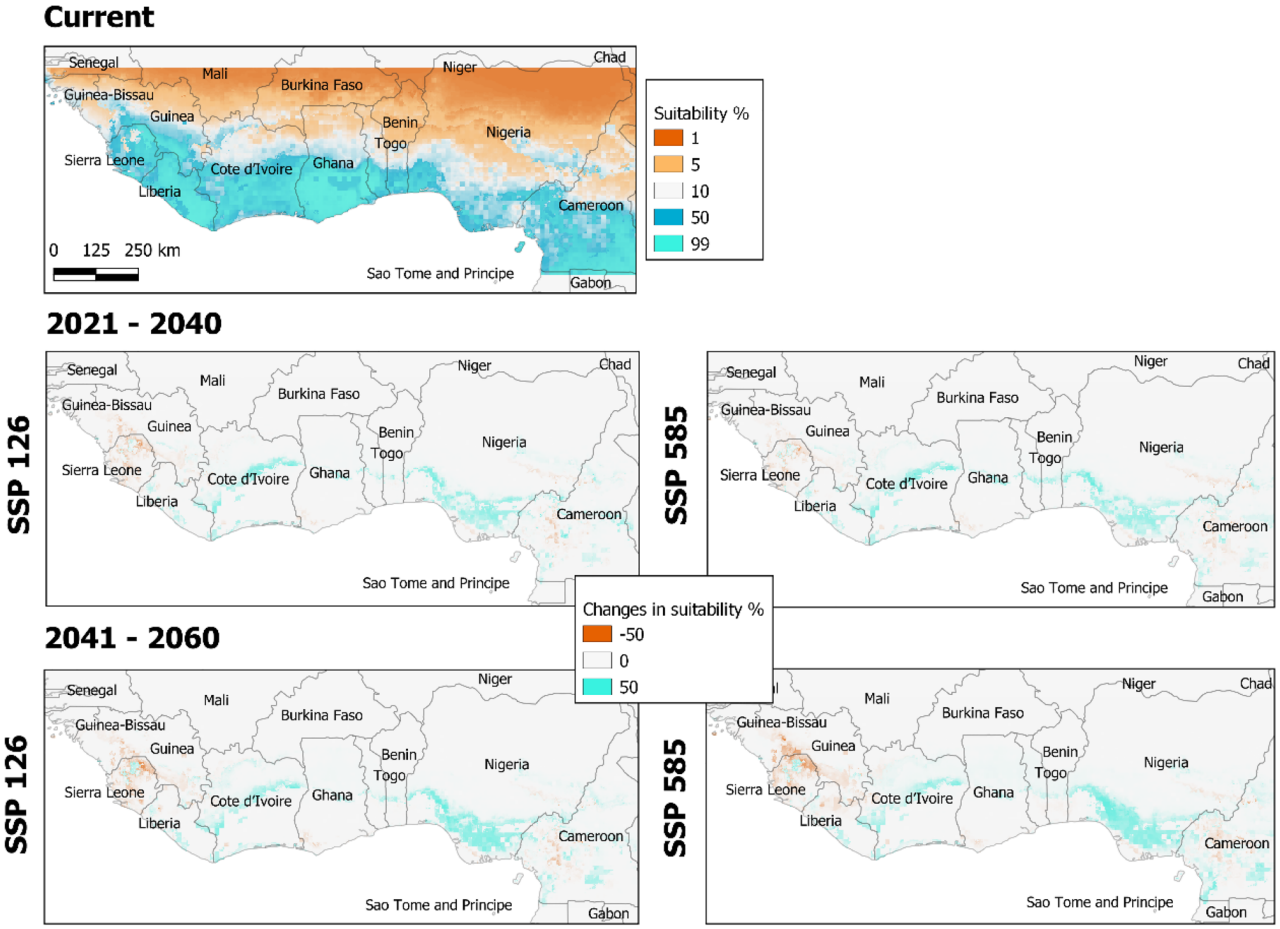
Relative climatic suitability for *Theobroma cacao* L. under current conditions (top), as well as shifts in suitability due to climate change by 2021–2040 (middle) and 2041–2060 (bottom) in West Africa, considering two Shared Socio-economic Pathways: SSP126 (left) and SSP585 (right), low and high emissions scenarios, respectively. Light blue indicate new potential areas for *Theobroma cacao* L. and orange indicates areas expected to be no longer suitable under projected climate conditions. Graphs were generated by QGIS 3.26.3 (https://www.qgis.org) with the global vector data from the GADM database (https://gadm.org) and the outputs of the suitability modelling.

We found for 2040 an increase of suitable area for *Theobroma cacao* L. ranging from 3.9% for the low emissions scenario and 4.8% for the high emissions scenario. Interestingly, by 2060, the increase will be of 0.2% (high emissions) and 6.7% (low emissions). This increase will be spatially heterogeneous across the region, with a reduction of suitable area in the West and increasing towards the East (Fig. 3).

**Figure 4:**
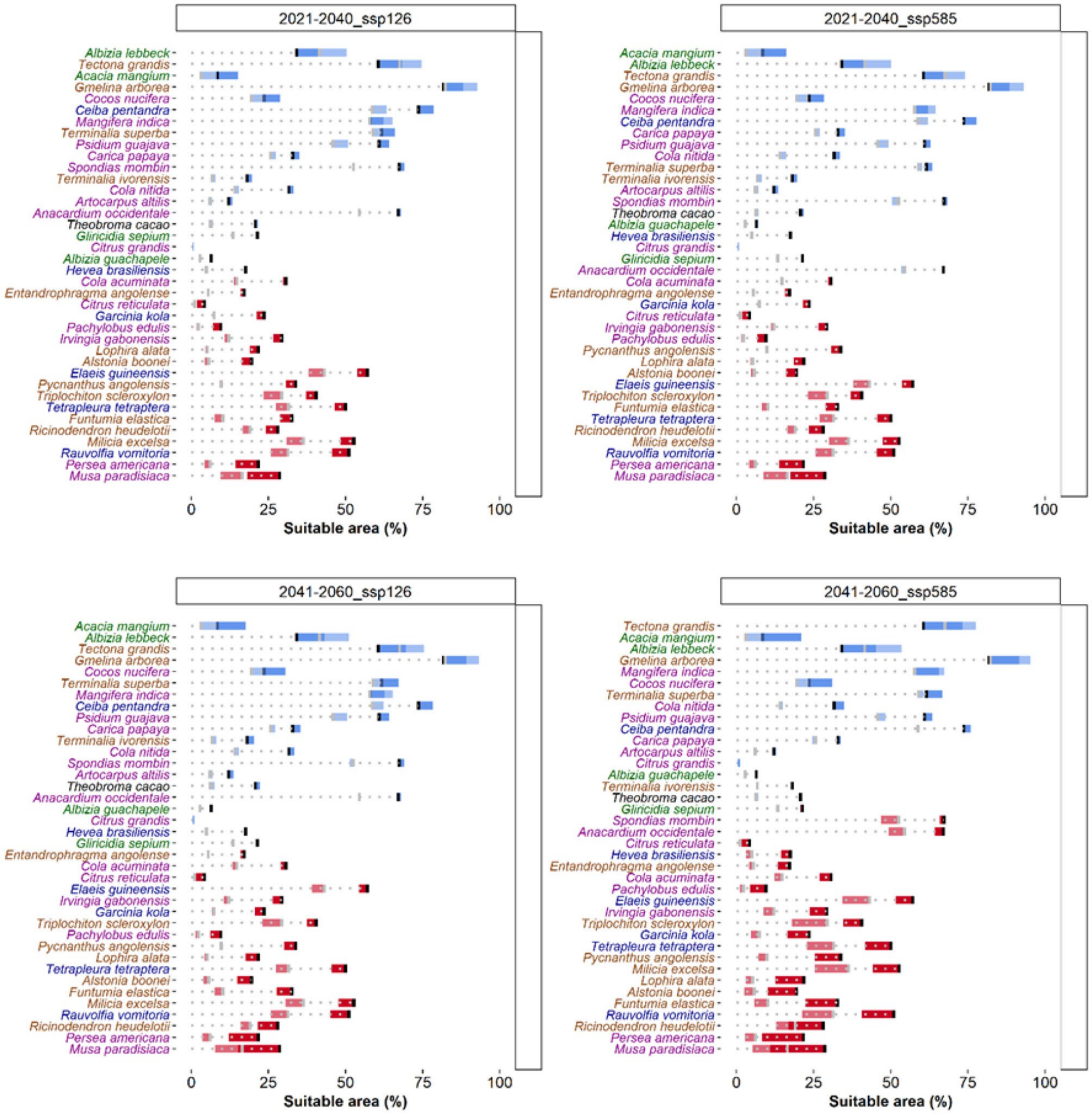
Change in suitable area for the 37 shaded tree species and cocoa considering two Shared Socio-economic Pathways: left SSP126 and right SSP585, low and high emissions scenarios, respectively, for the period 2021–2040 (top) and 2040–2060 (bottom). Black bars indicate the percentage of suitable area under the current climatic conditions. Blue bars show an increase in suitable area while red indicates decrease under future scenarios. Bars with darker colour indicate the percentage change considering all suitable area and bars with lighter colour change considering the area currently available for agroforestry not contributing to deforestation (i.e., shrubs cover areas, grassland, cropland, and sparse vegetation). Species are sorted from higher increase to higher decrease in suitable area. For species names: black for cocoa; brown for timber trees; green for leguminous trees; purple for fruit trees and blue for species with other uses.

Under a low emissions scenario (SSP126), 50% of the 37 shade tree species modelled will experience a decrease in geographic rate extent by 2040 in West Africa (Fig. 4). In the long term (2041–2060), 60% of shade tree species are projected to suffer a decrease in range extent within West Africa (Fig. 4). The widely used fruit species in C-AFS in West Africa, *Persea americana* Mill. and *Musa paradisiaca* L., are expected to lose up to a quarter of their range. Comparing SSP scenarios, changes were more pronounced under the SSP585 scenario relative to the SSP126 only for the long-term projection (Fig. 4). Several key shade species seem to be favoured in a future climate scenario. *Tectona grandis* L.f. and *Gmelina arborea* Roxb. ex Sm., used as a timber, and two leguminous trees (*Acacia mangium* Willd. and *Albizia lebbeck* (L.) Benth.) showed the highest increase of the distribution range (Fig. 4). Within the common fruit species currently growing in cocoa plantations *Mangifera indica* L., *Cocos nucifera* L., *Psidium guajava* L. and *Carica papaya* L. also expected an increase in geographic rate extent in West Africa (Fig. 4).

Percentage of suitable area per species was highly reduced when considering only the area currently available for agroforestry not contributing to deforestation (i.e., shrubs cover areas, grassland, cropland, and sparse vegetation). In fact, 92% of the species showed a reduction (Fig. 4) with cocoa showing a 14.5% reduction. In contrast, the relative changes in suitable area due to climate change scenarios were rather similar comparing values across all the study area and just the area not contributing to deforestation (Fig. 4).

**Figure 5:**
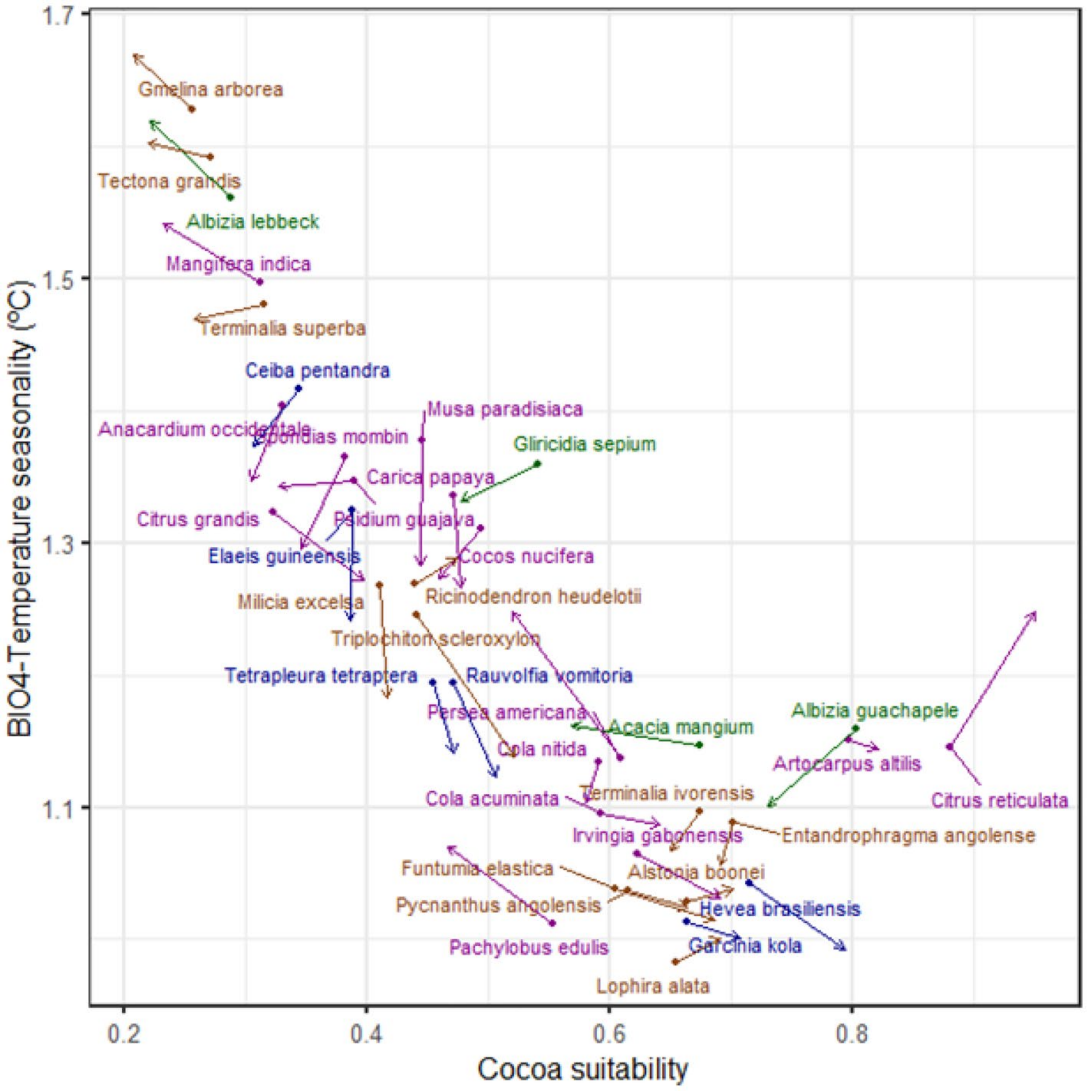
Mean cocoa habitat suitability and temperature seasonality for each species. Each dot corresponds to the mean seasonality and cocoa habitat suitability of the area suitable for the species under current climatic conditions, while the arrow indicates the shift for 2041- 2060 in the high emissions scenario (SSP585). Species towards the bottom right corner show low temperature seasonality affinity and high overlap with cocoa habitat suitability. In brown, timber trees; green, N-fixing trees; purple, fruit trees and blue, species with other uses.

We also calculated the mean cocoa habitat suitability and the most relevant variable across all species SDM (temperature seasonality) in the area projected as suitable for each species considering all the study area. The pool of shade species considered cover the range of temperature seasonality in the study area (Fig. 5), with a well-mix of main potential uses. Species adapted to more seasonal conditions (e.g., *Albizia lebbeck* (L.) Benth.) have in general lower overlap with cocoa suitable conditions (Fig. 5). This pattern seems to be exacerbated under future climatic conditions.

**Figure 6:**
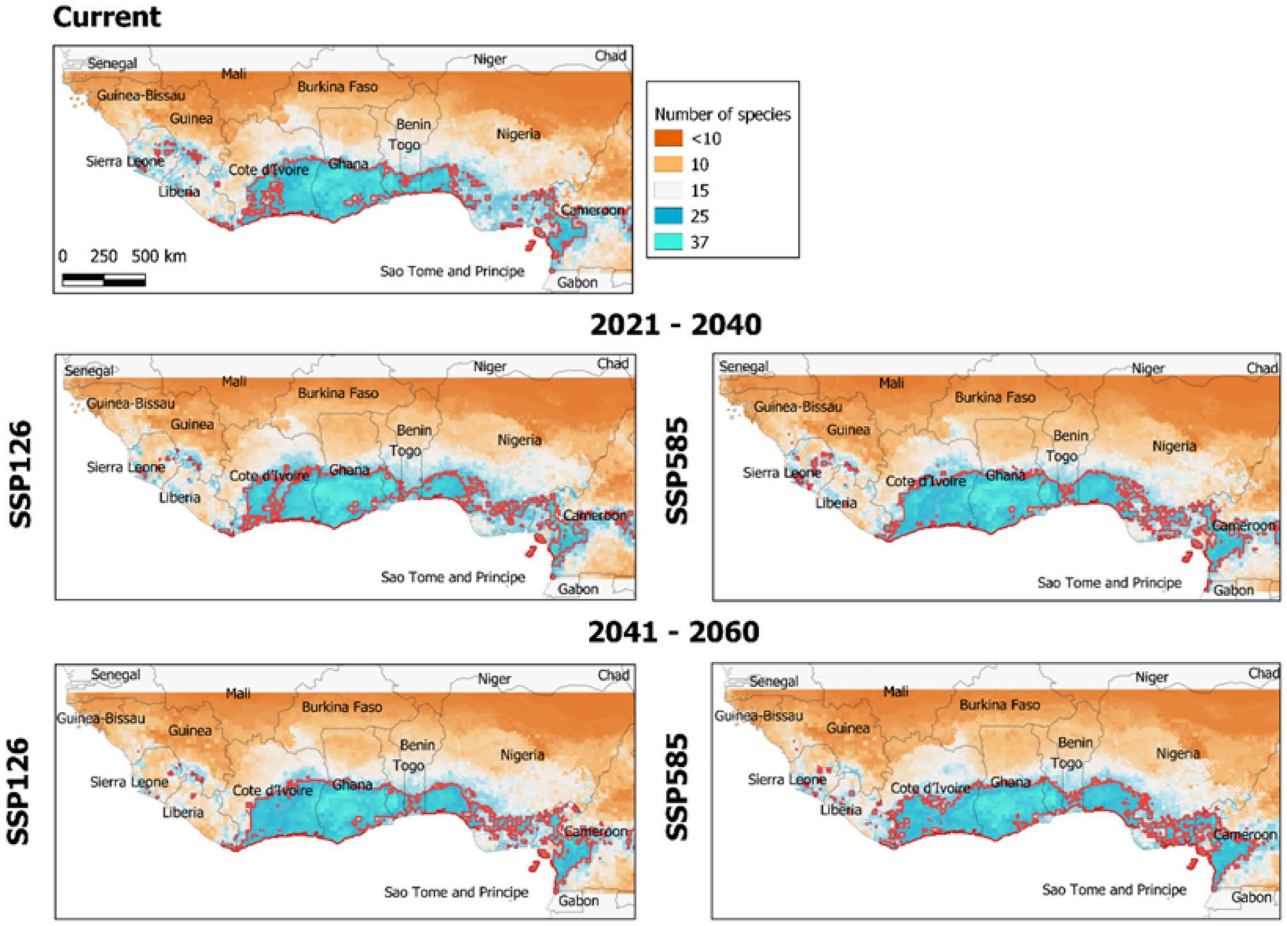
Current (top) and future projection of ensemble models for shade tree species richness (37 shaded tree species and cocoa) by 2021–2040 (middle) and 2041–2060 (bottom) in West Africa, considering two Shared Socio-economic Pathways: SSP126 (left) and SSP585 (right), low and high emissions scenarios, respectively. Light blue indicate areas with high species richness (SR) and orange indicates areas with low SR. The red lines show hotspots of SR (+ 25 species). Graphs were generated by QGIS 3.26.3 (https://www.qgis.org) with the global vector data from the GADM database (https://gadm.org) and the outputs of the suitability modelling.

Geographically, results obtained by ensemble models indicated that bioclimatic habitat of ~ 25 shade tree species overlaps with that of *Theobroma cacao* L. in the coastal zones of West Africa (Fig. 6; also see Fig. 3). Two major areas: south part of Côte d’Ivoire and Ghana and west part of Cameroon, were identified with the highest potential for C-AFS by 2060. In this period, approximately 70% of the cocoa distribution areas would be suitable for more than 25 shade tree species (Fig. 6).

## Discussion

Previous research has shown that the effects of climate change might compromise the sustainability of cocoa plantations in West Africa^4–6^. However, few modelling frameworks have incorporated potentially ecologically relevant sets of predictors beyond climate variables. To our best knowledge, this is the first time that edaphic variables have been incorporated into distribution models to study the potential impacts of climate change on cocoa-based agroforestry systems. In the present study, we attempt to identify those climatic and edaphic factors that are most likely to become limiting to cocoa and associated species and that must be considered when designing adaptation strategies in smart agroforestry. Our results suggest that land use planning that considers potential climate change impacts will be needed for agroforestry systems to remain as the best alternative for climate adaptation of cocoa fields.

Climate variables were the most important factor to explain the distribution of cocoa and most of the shade tree species considered for West Africa. Across species, the most important predictor was temperature seasonality with up to 80% relevance for some species (see Fig. 2). In contrast, precipitation variables (e.g., precipitation of the warmest and coldest quarters, and maximum monthly precipitation) were only relevant for certain species. Particularly for *Theobroma cacao* L., the contribution rate of temperature seasonality reached > 50%, while precipitation of the warmest quarter was the second most important. Considering this finding, the tolerance to variables temperatures should also be considered as an important factor in defining product profiles for cocoa breeding programs.

The distribution of native species was more constrained by climate than exotic species. Whether this means that exotic species can escape changing climate conditions better than native species is uncertain, but their range expansion in response to climate change is expected to be more restricted by the availability of suitable edaphic conditions. Our results show that soil pH is the most important edaphic variable across shade tree species. This result is consistent with recent research that emphasizes the importance of soil pH shaping species distribution^17^, since it affects nutrient availability, the release of toxic elements^20^ and bacterial communities^21^. The other factors considered (content of sand, nitrogen and CEC) were the least important variables shaping their distribution. These results could be explained by two confounded reasons. On one hand, the spatial resolution of edaphic variables was rather coarse affecting their ability to reflect relevant changes in soil conditions operating at lower scale (e.g. due to topographic variations). On the other hand, due to the large scale of this study we might expect climate is driving species distribution while other landscape and soil factors might be more relevant at smaller scales^22^. Other potentially important predictors that could limit the ability of plant species to establish in areas that have just entered their climatic niches, such as topography or socio-economic factors, are not included in the study. Future studies, could look into the importance of these relevant variables particularly at smaller scales.

According to our results, the potential habitat suitability for cocoa in West Africa would remain constant or slightly increase in the next decades. Interestingly the larger increase is predicted for the lower carbon emissions scenario (SSP126) by 2060, while it seems to remain constant for the high carbon emissions scenario (SSP585). This pattern could be the result of a non-linear relation of cocoa suitability with climate variables, suggesting that a moderate increase will be positive but that it will turn into negative under the more extreme scenario. This result rejects our basic initial hypothesis and contradicts previous studies carried out in West Africa where they forecasted a considerable reduction of suitability in the region^4–6^. Nevertheless, some of these findings are in line with those of Black et al. (2021), who found that in the regions where cocoa is currently grown in Ghana, Côte d’Ivoire and Togo, the Net Primary Productivity is projected either to stay the same or to increase slightly. Here, we used a comprehensive consensus approach from the global distribution of cocoa which is the widely accepted methodology to characterize the current potential distribution of species^23,24^. However, previous studies only considered data from Côte d’Ivoire and Ghana or constructed distribution maps based on expert knowledge^5^. The restricted dataset used in those studies limit their ability to predict the suitability of the species as it only considers a narrow climate variability. In addition, different from previous studies, we used the latest climate projections from the new CMIP6 models.

The projected increase in suitability occurs mostly in the central part of Côte d’Ivoire and south-eastern Nigeria but under marginal suitable levels (i.e., < 60%) which might threaten their sustainability. This is a consequence of a change in the spatial pattern of precipitation (mainly the precipitation of warmest quarter). While the projected average values for the whole region remain close to the current values, models predict strong changes in specific regions (see supplementary material Tables S5, S6 and Fig. S16).

Conversely, our model projections show a severe reduction in habitat suitability of the currently highly suitable areas of cocoa in the western (i.e., Guinea, Sierra Leone, and Liberia), which may even turn unsuitable by 2060 (see Fig. 3). This pattern is also predicted by Schroth et al. (2016) with a reduction in 20–30% in suitability for Sierra Leone. This is due to an increase in maximum temperatures in this region and the water balance disruption of the dry season, with losses of up to 50% of precipitation in the warmest quarter. Consequently, current cocoa plantations in these areas will likely undergo extreme climate conditions requiring further inputs such as irrigation or smart shade management^25^.

In this study, we projected future scenarios of climate change, assuming that edaphic variables and land use patterns remain constant. However, climate change and land degradation can change land-use patterns and soils dramatically^26^, producing a synergistic effect on species distributions. Although future scenarios of land-use change are rarely available, futures studies might look at this interactive effect of driving forces, particularly given that current agroforestry practices may reduce the likelihood of soil erosion and degradation^27^.

Under expected climate change scenarios, marginal suitable areas for cocoa production will require optimum shade management to protect cocoa trees from high temperatures^28^. Cocoa-farmers can make use of a wide variety of shade and agroforestry tree species with different additional benefits (e.g., timber, N-fixing, and fruits). However, each species has environmental requirements that could be limiting both under current and future climate scenarios. Approximately half of the shade tree species considered in this study showed an increase in suitable area across the region for the expected climate scenarios in 2040 and 2060 (see Figs. 4, 5). Particularly, teak (*Tectona grandis* L.f.), a widely used timber species, is among the species that will largely benefit from the future warmer conditions, as observed in other geographical areas^29^. Considering its relevance as timber species across the tropics, these findings encourage its wide use in agroforestry systems. On the other hand, the models project a considerable shrink in suitable area for several fruit species widely used in West Africa (e.g., *Musa paradisiaca* L. and *Persea americana* Mill.) which might require the shift to alternative species with less bioclimatic limitations and a potential increase in suitability area, such as *Mangifera indica* L. or *Carica papaya* L.

The variety of shade species that farmers could use in the design of their agroforestry systems is highly context dependent based on the environmental and economic limitations. The pool of species considered show a well coverage across the main environmental gradient in the region (i.e. temperature seasonality), including an adequate mix of different main uses. Nevertheless, the choice of N-fixing species is very limited at the lower range of temperature seasonality. Given the importance of these species to ameliorate soil conditions it might be relevant to identify other potential candidates (e.g., *Pterocarpus erinaceus* Poir. or *Leucaena diversifolia* (Schltdl.) Benth.). Overall, within each range of temperature seasonality there are shade species with at least partial overlap with cocoa habitat suitability (> 0.2). Thus, it seems farmers have a wide variety of shade species that could be selected according to the environmental, and economic context maximizing the cocoa habitat overlap of their locality.

Percentage of suitable area per species was highly reduced when considering only the area currently available for agroforestry not contributing to deforestation. This finding indicates that the feasibility of establishment of new cocoa agroforestry plantations in currently non-forested areas might be limited. In fact, only 6% of this area might be suitable for cocoa (~ 67,000 km^2^). Considering that current surface devoted to cocoa in the region is 77,556 km^2^^30^, this suitable space although limited might be of special importance to reduce the pressure of intensive cocoa farming to forests.

Geographically, southern Ghana and Côte d’Ivoire seem to be the current hotspot of shade species richness (> 30 species with high environmental suitability; see Fig. 6), with also some high richness pockets in Nigeria and Cameroon. Among other reasons, these clusters could be the result of cocoa production intensity across this central region. The pre-selection of shade species in this study is the consensus of the species commonly used across the countries in West Africa^31^, yet this list may be biased towards species used in the two main cocoa-producers countries, Ghana and Côte d’Ivoire. From these countries, the crop management and tree selection could have been easily exported across the West Africa region. Interestingly, those countries located further away from the hotspot of cocoa production, Liberia and Cameroon, showed low variability of shade tree species (10–20 species), despite showing high levels of cocoa environmental suitability. Enhancing cocoa production in agroforestry systems in these countries will require the selection of much better-adapted tree shade species to the future environmental conditions, likely from their rich native flora^32^. Future scenarios show a reduction of shade species richness, particularly in Côte d’Ivoire. This trend is especially relevant under the more extreme socioeconomic pathway. Despite the reduction in the variety of species that farmers could use, it is relevant that all cocoa producing areas will at least keep more than 10 species under environmental suitable conditions.

From the list of shade tree species commonly used in West Africa^31^, around half of them were exotic species. Scientific advancements in plant sciences and agroforestry technologies have helped expand the range of several of these species worldwide^33^. Examples of trees and plants include *Acacia* spp., *Gliricidia sepium* (Jacq.) Kunth, *Tectona grandis* L.f. and *Musa* spp. This is partly due to the fact that native tree systems rank very low in the list of recommendations of developmental agencies, probably due to a lack of knowledge of their distribution, management and potential^34^. More in-depth scientific investigations and better communications on the effect of different native tree species on yield and provisioning ecosystem services is hence required to motivate their selection in agroforestry systems.

While there are good conceptual and empirical arguments supporting our conclusions, modelling species distribution in space and time is based on assumptions inherent in the models^35^. Thus, there are certain limitations and gaps in the data to effectively apply the modelling approach. First, we used interpolated climate and soil grids relying on spatial and topographic predictors (WorldClim and SoilGrids) which are unlikely to capture complex spatial features of the climate and soil and therefore add their own uncertainty to our models^36^. However, whilst the quantitative results could be quite different using field data, we believe that the overall projections and trends over the large study area considered here will be similar with the present resources at hand. Second, the area highly suitable for the different species obtained from this work does not necessarily translate into a measure of successful field establishment, as in addition to the environmental factors used in this study, multiple other physical and socioeconomic factors might determine their survival (e.g. management). Despite these challenges, the results obtained from these models are a starting point to adapt most of the cocoa production systems in West Africa to climate change. Finally, as new modelling approaches and datasets are developed, there will be a need to re-analyse the existing data, with greater temporal and spatial resolution.

## Materials and methods

### Selection of tree species

We compiled a list of shade tree species based on farmers preference and recommended tree species in cocoa growing systems of West Africa^31^. The list includes 45 species commonly used as shade trees in Ghana, Côte d’Ivoire, Cameroon, and Nigeria. From these 45 species, 18 are mainly used as food (fruit, seed, flower, or nut), 15 species mainly used for timber products (firewood or wood) and 12 species are commonly used for other uses (oil, latex, or fiber) (supplementary material Table S1). This list includes a few species commonly used for their potential to improve soil conditions by fixing nitrogen, such as *Gliricidia sepium* (Jacq.) Kunth, *Acacia mangium* Willd., or *Albizia lebbeck* (L.) Benth. From these 45 species, 25 are native to West Africa and 20 are introduced exotic species (supplementary material Table S1).

### Occurrence dataset

We obtained for all 45 species, a total of 33,895 occurrence points from the Global Biodiversity Information Facility (GBIF) (https://www.gbif.org) at global scale (list of all downloads used in supplementary material Table S1).

Unsuitable presence locations points were identified and deleted in a step-by-step procedure, according to the following criteria: records (i) with no geographic information or with incomplete coordinates; (ii) with clear errors such as locations in the ocean and mismatches between administrative data and coordinates; (iii) collected before 1969 to meet the current baseline climate used; (iv) in marginal climates (e.g., botanical gardens in temperate climate) within the last 10% of the climatic gradient distribution and; (v) “fossil” records or from unknown sources. We also reduced the possible effects of sampling bias and spatial autocorrelation keeping only one record per species per 2.5 arc-min grid (to overlap with environmental information). Finally, we selected those species with *n* > 60 presence locations points. The final dataset included 14,368 occurrences for 38 shade tree species and 401 locations points for cocoa.

The algorithms used in this study require absence data for calibration and validation of the model, but reliable absence information is rarely available. Thus, we generated 1000 pseudoabsences per species randomly drawn from an area less than 500 km from the occurrence points per species. This buffer area was a good compromise to identify distinct environmental conditions without overfitting results^37^.

### Environmental data compilation and pre-processing

Climate information was extracted from the WorldClim-Global Climate Database^38^ (https://worldclim.org), reporting gridded mean climate values from a baseline period of 1970–2000. The dataset was downloaded globally at 2.5 arc-min spatial resolution (~ 5 × 5 km at the equator). The dataset considered 19 climate variables commonly used in biogeographical studies. Outputs from the new circulation models of the Coupled Model Inter-Comparison Project Phase (CMIP6) were used to assess projected changes in future climate. Downscaled monthly future climate data from CMIP6 is available for nine global climate models, and four Shared Socio-economic Pathways (SSPs). To provide plausible future scenarios for management, we selected two contrasting SSPs scenarios (i.e., SSP126 and SSP585), and the two first periods (i.e., 2021–2040, 2041–2060).

Soil data was extracted from SoilGrids database^39^ (https://soilgrids.org), which provides maps for ten different soil properties at six different depths. SoilGrids prediction models are fitted using soil profile observations, and other environmental information including climate, land cover and terrain morphology. The download of selected SoilGrids layers (Table 1) was performed with Google Earth Engine (GEE) using the rgee package^40^. In this work, we used the 5 km resolution files averaged for layers from 30 cm to 1 m depth as it represents most relevant horizon for trees independent of soil management. To be conservative, we assumed that edaphic variables remain constant under future scenarios^41^.

**Table 1:** List of the main environmental variables for species distribution model of cocoa and associated shade tree species in West Africa.

Variable	Code	Units
WorldClim		
Annual mean temperature	BIO1	°C
*Mean Diurnal Range (Mean of monthly (max temp—min temp))*	***BIO2***	°C
*Isothermality (BIO2/BIO7) (*× *100)*	*BIO3*	
*Temperature Seasonality (standard deviation* × *100)*	***BIO4***	
Max temperature of warmest month	BIO5	°C
Min Temperature of Coldest Month	BIO6	°C
Temperature annual range (BIO5–BIO6)	BIO7	°C
*Mean Temperature of Wettest Quarter*	***BIO8***	°C
*Mean Temperature of Driest Quarter*	***BIO9***	°C
Mean Temperature of Warmest Quarter	BIO10	°C
Mean Temperature of Coldest Quarter	BIO11	°C
Annual precipitation	BIO12	mm
*Precipitation of Wettest Month*	***BIO13***	mm
*Precipitation of driest month*	*BIO14*	mm
*Precipitation Seasonality (Coefficient of Variation)*	***BIO15***	mm
Precipitation of Wettest Quarter	BIO16	mm
Precipitation of Driest Quarter	BIO17	mm
*Precipitation of Warmest Quarter*	***BIO18***	mm
*Precipitation of Coldest Quarter*	***BIO19***	mm
SoilGrids		
*Bulk density*	*bdod*	Cg/cm^3^
*Clay content*	*clay*	g/kg
*Sand*	***sand***	g/kg
*Silt*	*silt*	g/kg
*Cation exchange capacity (at ph 7)*	***cec***	Mmol(c)/kg
*Nitrogen*	***nitrogen***	Cg/kg
*pH water*	***phh2o***	pH*10
World Reference Base (2006) Soil Groups*	wrb	

*Legend in: https://soilgrids.org/

In italics, chosen variables by VIF analysis. In bold italic type, final variables selected by correlation analysis.

The environmental dataset was reduced by analysis of collinearity using the Variance Inflation Factor (VIF). The predictor variables with VIF > 8 were removed^42^. Then, a correlation matrix using Pearson’s correlation coefficient was calculated and variables with (r > ǀ0.7ǀ) were removed. Final selection between correlated variables was decided according to higher ecological meaning for tropical and subtropical species based on expert recommendation. The original 27 environmental variables were reduced to twelve non-collinear variables (Table 1).

### Species distribution modelling

Suitability modelling and mapping of all species were done in R^43^ using a consensus method for species distribution modelling (SDM) compiled by the package BiodiversityR^44^. We selected four algorithms commonly used in SDM (supplementary material Table S2).

We performed a fourfold cross-validation per species by randomly assigning location data to four bins. The SDM performance was assessed by the Area Under the Curve (AUC) criterion and True Skill Statistic (TSS)^45^. For each species and algorithm, we calculated a weight as the average of the AUC of the different algorithms of the four runs. Then, an ensemble (i.e. consensus) output for the current potential distribution of each species was calculated as the weighted average of different algorithms^46^. Considering that each algorithm might provide a potential plausible outcome, an ensemble model combines the uncertainty across them yielding more accurate estimates^47^. The AUC and TSS values of the selected SDM models and the ensemble model are shown in supplementary material Table S3. The relative importance (%) of each environmental variable was estimated per species according to the best algorithm. We also tested whether the accumulated relative importance between climatic and edaphic variables varied across the main use and the origin of the species using post-hoc Tukey tests in a linear regression using the R package multcomp^48^. All data processing and model development was carried out using the R package *BiodiversityR* based on the workflow by^13^.

### Model projections and outputs

As a study area, we have delimited a 2.904.160 km^2^ band of land covering the total area of the main cocoa-producing countries in West Africa (Côte d'Ivoire, Ghana, Nigeria, Cameroon, Sierra Leone, Liberia, Togo, Benin, Guinea and Guinea-Bissau). However, suitable habitats may be occupied or unusable for the establishment of cocoa agroforestry systems. For example, artificial surfaces, water bodies, flood zones, and primary forests can hardly be transferred to cocoa plantations. For this reason, in order to select the areas that can be actually used for agriculture, it was necessary to constrain the projections obtained from SDMs by taking into account certain parameters, such as land cover. Thus, suitable area was estimated for all the study area and for the area strictly available for cocoa agroforestry avoiding deforestation (~ 1.100.000 km^2^, including shrubs cover areas, grassland, cropland and sparse vegetation). Land-cover information was obtained from the European Space Agency based on the Copernicus Sentinel-2A images at 20 m (http://www.esa.int/ESA_Multimedia/Images/2017/10/African_land_cover). The land cover classification map was created between December 2015 and December 2016.

Ensemble models per species calibrated with current climate were projected into future climate scenarios according to seven global climate models (GCMs) and two contrasting SSPs to predict the distribution of suitability by the 2021–2040 and 2041–2060 periods. In these projections, we assumed land-cover remained stable according to current conditions (2016) as projection for land-use change was not available in the study area. Continuous predictions were transformed into binary results per species. This transformation was applied using an optimization procedure that maximizes the sensitivity and specificity of the output^49^. These binary maps were used to estimate the change in suitable area available per species, SSPs and period.

To identify the overlap in habitat suitability between each species and cocoa across the most relevant environmental gradient, we calculated per shade tree species within their area projected as suitable (a) the mean cocoa habitat suitability and (b) the mean of the most relevant variable for all species. These averages were estimated for current conditions and the SSP585 by 2041–2060. Finally, shade species richness maps for the study area were calculated per SSP and time-period accumulating the binary results across all shade species.

## Supplementary Information

Supplementary Information.

## Data Availability

Data and R code used is available through Dataverse^50^. The full project replication workflow is available through GitHub https://github.com/Ariza123/WP5_C-AFSsuitability.
