# Supplementary material for: Vulnerability of cocoa-based agroforestry systems to climate change in West Africa
Source: Sci Rep. 2023 Jun 20;13:10033. doi: 10.1038/s41598-023-37180-3 (PMC10281996; doi:10.1038/s41598-023-37180-3)
Supplement: Supplementary file 1 — Supplementary Information. [file 41598_2023_37180_MOESM1_ESM.docx]

**Supplementary Information for**

**Vulnerability of cocoa-based agroforestry systems to climate change in West Africa**

Antonio Jesús Ariza Salamanca, Rafael Mª Navarro-Cerrillo, José L. Quero-Pérez, Belinda Gallardo-Armas, Jayne Crozier, Clare Stirling, Kauê de Sousa, Pablo González-Moreno

**Correspondence to:**

Antonio Jesús Ariza Salamanca

**Email:** o32arsaa@uco.es

**This PDF file includes:**

**Tables S1 to S6**

**Figures S1 and S17**

**TABLES**

Table S1. Tree species according to their overall frequency in cocoa plantations: complete botanical names and local names, uses, origin and GBIF doi.

| Scientific names | Local names | Uses | Origin | GBIF DOI |
| --- | --- | --- | --- | --- |
| *Persea americana* Mill. | avocat | Fruit | Exotic | GBIF.org (06 October 2020) GBIF Occurrence Download https://doi.org/10.15468/dl.grwygx |
| *Citrus reticulata* Blanco | mandarine | Fruit | Exotic | GBIF.org (14 October 2020) GBIF Occurrence Download https://doi.org/10.15468/dl.ys67my |
| *Mangifera indica* L. | amango | Fruit | Exotic | GBIF.org (14 October 2020) GBIF Occurrence Download https://doi.org/10.15468/dl.8qydf3 |
| *Citrus grandis* (L.) Osbeck | orange | Fruit | Exotic | GBIF.org (14 October 2020) GBIF Occurrence Download https://doi.org/10.15468/dl.rhgnh9 |
| *Citrus sinensis* (Mill.) Pers., 1806 | orange | Fruit | Exotic | GBIF.org (14 October 2020) GBIF Occurrence Download https://doi.org/10.15468/dl.pxpete |
| *Cola nitida* (Vent.) Schott & Endl. | wese | Fruit | Native | GBIF.org (14 October 2020) GBIF Occurrence Download https://doi.org/10.15468/dl.sq48b3 |
| *Cola acuminata* (P.Beauverd) Schott & Endl. | Kola | Fruit | Native | GBIF.org (14 October 2020) GBIF Occurrence Download https://doi.org/10.15468/dl.escud9 |
| *Cocos nucifera* L. | kpako | Fruit | Exotic | GBIF.org (14 October 2020) GBIF Occurrence Download https://doi.org/10.15468/dl.pg826n |
| *Acacia mangium* Wild. | mangium | N-fixing | Exotic | GBIF.org (14 October 2020) GBIF Occurrence Download https://doi.org/10.15468/dl.ruxuux |
| *Anacardium occidentale* L. | cajou | Fruit | Exotic | GBIF.org (14 October 2020) GBIF Occurrence Download https://doi.org/10.15468/dl.pbnx8v |
| *Artocarpus altilis* (Parkinson) Fosberg | pa | Fruit | Exotic | GBIF.org (14 October 2020) GBIF Occurrence Download https://doi.org/10.15468/dl.5qrvrr |
| *Carica papaya* L. | papaye | Fruit | Exotic | GBIF.org (14 October 2020) GBIF Occurrence Download https://doi.org/10.15468/dl.r95wm8 |
| *Gliricidia sepium* (Jacq.) Kunth | gliricidia | N-fixing | Exotic | GBIF.org (06 October 2020) GBIF Occurrence Download https://doi.org/10.15468/dl.v79v5n |
| *Gmelina arborea* Roxb. ex Sm. | Gamhar | Timber | Exotic | GBIF.org (14 October 2020) GBIF Occurrence Download https://doi.org/10.15468/dl.9tkq25 |
| *Hevea brasiliensis* (Willd. ex A.Juss.) Müll.Arg. | shiringa | Others | Exotic | GBIF.org (14 October 2020) GBIF Occurrence Download https://doi.org/10.15468/dl.qp5ff6 |
| *Irvingia gabonensis* (Aubry-Lecomte ex O'Rorke) Baill. | dika | Fruit | Native | GBIF.org (14 October 2020) GBIF Occurrence Download https://doi.org/10.15468/dl.3kgds5 |
| *Musa paradisiaca* L. | plantain | Fruit | Exotic | GBIF.org (06 October 2020) GBIF Occurrence Download https://doi.org/10.15468/dl.86mgmv |
| *Psidium guajava* L. | guayabo | Fruit | Exotic | GBIF.org (14 October 2020) GBIF Occurrence Download https://doi.org/10.15468/dl.86jrt9 |
| *Tectona grandis* L.f. | teck | Timber | Exotic | GBIF.org (06 October 2020) GBIF Occurrence Download https://doi.org/10.15468/dl.3xzy5b |
| *Markhamia lutea* (Benth.) K.Schum. | botoro | Others | Exotic | GBIF.org (14 October 2020) GBIF Occurrence Download https://doi.org/10.15468/dl.k4436n |
| *Elaeis guineensis* Jacq. | me | Others | Native | GBIF.org (14 October 2020) GBIF Occurrence Download https://doi.org/10.15468/dl.umugb3 |
| *Ricinodendron heudelotii* (Baill.) Heckel | akpi | Timber | Native | GBIF.org (06 October 2020) GBIF Occurrence Download https://doi.org/10.15468/dl.p749pf |
| *Alstonia boonei* de Wild. | nyamedua | Timber | Native | GBIF.org (14 October 2020) GBIF Occurrence Download https://doi.org/10.15468/dl.74jzgc |
| *Funtumia elastica* (Preuss) Stapf | funtum | Timber | Native | GBIF.org (14 October 2020) GBIF Occurrence Download https://doi.org/10.15468/dl.wze5a9 |
| *Lophira alata* Banks ex C.F.Gaertn. | kaku | Timber | Native | GBIF.org (14 October 2020) GBIF Occurrence Download https://doi.org/10.15468/dl.7etxgf |
| *Terminalia ivorensis* A.Chev. | emire | Timber | Native | GBIF.org (14 October 2020) GBIF Occurrence Download https://doi.org/10.15468/dl.24dwm2 |
| *Terminalia superba* Engl. & Diels | ofram | Timber | Native | GBIF.org (14 October 2020) GBIF Occurrence Download https://doi.org/10.15468/dl.935wev |
| *Rauvolfia vomitoria* Afzel. | kakapenpen | Others | Native | GBIF.org (14 October 2020) GBIF Occurrence Download https://doi.org/10.15468/dl.pj2mdg |
| *Tetrapleura tetraptera* (Schumach. & Thonn.) Taub. | prekese | Others | Native | GBIF.org (14 October 2020) GBIF Occurrence Download https://doi.org/10.15468/dl.9qh4rx |
| *Dacryodes edulis* (G.Don) H.J.Lam | safoutier | Fruit | Native | GBIF.org (14 October 2020) GBIF Occurrence Download https://doi.org/10.15468/dl.bbc8u2 |
| *Khaya ivorensis* A.Chev. | mahogany | Timber | Native | GBIF.org (06 October 2020) GBIF Occurrence Download https://doi.org/10.15468/dl.65qkn2 |
| *Lovoa trichilioides* Harms | dibetou | Timber | Native | GBIF.org (14 October 2020) GBIF Occurrence Download https://doi.org/10.15468/dl.cw2ans |
| *Spondias mombin* L. | troma | Fruit | Exotic | GBIF.org (14 October 2020) GBIF Occurrence Download https://doi.org/10.15468/dl.ctusq3 |
| *Albizia lebbeck* (L.) Benth. | awiamfo | N-fixing | Exotic | GBIF.org (14 October 2020) GBIF Occurrence Download https://doi.org/10.15468/dl.mnc83j |
| *Albizia guachapele* (Kunth) Dugand | semina | N-fixing | Exotic | GBIF.org (14 October 2020) GBIF Occurrence Download https://doi.org/10.15468/dl.nz75jb |
| *Triplochiton scleroxylon* K.Schum. | wawa | Timber | Native | GBIF.org (14 October 2020) GBIF Occurrence Download https://doi.org/10.15468/dl.errvvc |
| *Garcinia kola* Heckel | ngadiadia | Others | Native | GBIF.org (14 October 2020) GBIF Occurrence Download https://doi.org/10.15468/dl.uux5mw |
| *Milicia excelsa* (Welw.) C.C.Berg | odum | Timber | Native | GBIF.org (14 October 2020) GBIF Occurrence Download https://doi.org/10.15468/dl.sjkxzk |
| *Pycnanthus angolensis* (Welw.) Exell | otie | Timber | Native | GBIF.org (14 October 2020) GBIF Occurrence Download https://doi.org/10.15468/dl.m7tgu5 |
| *Ceiba pentandra* (L.) Gaertn. | onyina | Others | Exotic | GBIF.org (14 October 2020) GBIF Occurrence Download https://doi.org/10.15468/dl.xzr2rf |
| *Entandrophragma angolense* (Welw.) C.DC. | edinam | Timber | Native | GBIF.org (14 October 2020) GBIF Occurrence Download https://doi.org/10.15468/dl.8askkt |
| *Baillonella toxisperma* Pierre | moabi | Timber | Native | GBIF.org (14 October 2020) GBIF Occurrence Download https://doi.org/10.15468/dl.64kpz6 |
| *Entandrophragma cylindricum* (Sprague) Sprague | sapele | Timber | Native | GBIF.org (14 October 2020) GBIF Occurrence Download https://doi.org/10.15468/dl.ufn8qw |
| *Mansonia altissima* A.Chev. | bete | Timber | Native | GBIF.org (14 October 2020) GBIF Occurrence Download https://doi.org/10.15468/dl.p9q463 |
| *Theobroma cacao* L. | cocoa | Fruit | Exotic | GBIF.org (06 October 2020) GBIF Occurrence Download https://doi.org/10.15468/dl.p4c68k |

Table S2. Algorithms selected for (ensemble) species distribution modelling of cocoa and associated shade tree species in West Africa.

| Model | Reference |
| --- | --- |
| Generalized Boosted Regression Models (GBM) | Greenwell *et al*., 2019 |
| Generalized Additive Models (GAM) | Guisan *et al*., 2002 |
| Generalized Linear Models (GLM) | Guisan *et al*., 2002 |
| Random Forest (RF) | Breiman, 2001 |

Table S3. Summary of the statistical criteria computed for the valuation of SDMs. Area Under the Curve (AUC) and True Skills Statistics (TSS).

| **Species** | **GBM** | | **GLM** | | **GAM** | | **RF** | | **ENSEMBLE** | |
| --- | --- | --- | --- | --- | --- | --- | --- | --- | --- | --- |
|  | **AUC** | **TSS** | **AUC** | **TSS** | **AUC** | **TSS** | **AUC** | **TSS** | **AUC** | **TSS** |
| *Acacia mangium* | 0.94 | 0.75 | 0.93 | 0.73 | 0.90 | 0.69 | 0.82 | 0.61 | 0.93 | 0.70 |
| *Albizia guachapele* | 0.97 | 0.86 | 0.97 | 0.89 | 0.96 | 0.85 | 0.93 | 0.84 | 0.97 | 0.87 |
| *Albizia lebbeck* | 0.95 | 0.77 | 0.93 | 0.79 | 0.85 | 0.58 | 0.91 | 0.81 | 0.94 | 0.82 |
| *Alstonia boonei* | 0.96 | 0.81 | 0.85 | 0.70 | 0.91 | 0.70 | 0.84 | 0.65 | 0.95 | 0.80 |
| *Anacardium occidentale* | 0.90 | 0.72 | 0.89 | 0.69 | 0.89 | 0.66 | 0.86 | 0.71 | 0.90 | 0.71 |
| *Artocarpus altilis* | 0.90 | 0.72 | 0.89 | 0.79 | 0.92 | 0.77 | 0.90 | 0.75 | 0.95 | 0.76 |
| *Carica papaya* | 0.91 | 0.70 | 0.91 | 0.69 | 0.82 | 0.49 | 0.86 | 0.70 | 0.91 | 0.70 |
| *Ceiba pentandra* | 0.95 | 0.78 | 0.93 | 0.74 | 0.87 | 0.60 | 0.91 | 0.81 | 0.95 | 0.79 |
| *Citrus grandis* | 0.91 | 0.73 | 0.89 | 0.72 | 0.86 | 0.61 | 0.84 | 0.60 | 0.91 | 0.73 |
| *Citrus reticulata* | 0.85 | 0.64 | 0.84 | 0.59 | 0.78 | 0.54 | 0.78 | 0.57 | 0.85 | 0.68 |
| *Cocos nucifera* | 0.92 | 0.73 | 0.91 | 0.72 | 0.90 | 0.68 | 0.85 | 0.66 | 0.93 | 0.70 |
| *Cola acuminata* | 0.97 | 0.81 | 0.97 | 0.83 | 0.96 | 0.86 | 0.91 | 0.73 | 0.98 | 0.88 |
| *Cola nitida* | 0.99 | 0.95 | 0.96 | 0.93 | 0.96 | 0.84 | 0.96 | 0.92 | 1.00 | 0.94 |
| *Elaeis guineensis* | 0.98 | 0.87 | 0.97 | 0.87 | 0.94 | 0.77 | 0.96 | 0.89 | 0.98 | 0.88 |
| *Entandrophragma angolense* | 0.98 | 0.88 | 0.91 | 0.82 | 0.91 | 0.78 | 0.92 | 0.79 | 0.98 | 0.86 |
| *Funtumia elastica* | 0.98 | 0.90 | 0.85 | 0.71 | 0.90 | 0.75 | 0.90 | 0.72 | 0.97 | 0.86 |
| *Garcinia kola* | 0.97 | 0.89 | 0.87 | 0.75 | 0.85 | 0.71 | 0.97 | 0.93 | 0.98 | 0.92 |
| *Gliricidia sepium* | 0.98 | 0.85 | 0.97 | 0.84 | 0.93 | 0.75 | 0.94 | 0.87 | 0.98 | 0.87 |
| *Gmelina arborea* | 0.89 | 0.68 | 0.87 | 0.62 | 0.80 | 0.58 | 0.69 | 0.37 | 0.89 | 0.66 |
| *Hevea brasiliensis* | 0.92 | 0.73 | 0.93 | 0.73 | 0.90 | 0.69 | 0.82 | 0.64 | 0.92 | 0.74 |
| *Irvingia gabonensis* | 0.98 | 0.89 | 0.90 | 0.81 | 0.97 | 0.87 | 0.95 | 0.90 | 0.99 | 0.88 |
| *Lophira alata* | 0.98 | 0.95 | 0.97 | 0.93 | 0.92 | 0.79 | 0.91 | 0.83 | 0.99 | 0.94 |
| *Mangifera indica* | 0.93 | 0.72 | 0.92 | 0.69 | 0.80 | 0.53 | 0.91 | 0.79 | 0.93 | 0.78 |
| *Milicia excelsa* | 0.98 | 0.90 | 0.91 | 0.83 | 0.92 | 0.76 | 0.97 | 0.89 | 0.98 | 0.89 |
| *Musa paradisiaca* | 0.93 | 0.75 | 0.90 | 0.72 | 0.84 | 0.56 | 0.90 | 0.75 | 0.93 | 0.76 |
| *Pachylobus edulis* | 0.99 | 0.93 | 0.88 | 0.77 | 0.96 | 0.83 | 0.90 | 0.80 | 0.98 | 0.88 |
| *Persea americana* | 0.95 | 0.75 | 0.93 | 0.71 | 0.86 | 0.63 | 0.91 | 0.80 | 0.94 | 0.76 |
| *Psidium guajava* | 0.92 | 0.70 | 0.89 | 0.68 | 0.82 | 0.54 | 0.90 | 0.74 | 0.91 | 0.71 |
| *Pycnanthus angolensis* | 0.98 | 0.88 | 0.90 | 0.79 | 0.93 | 0.79 | 0.95 | 0.90 | 0.98 | 0.90 |
| *Rauvolfia vomitoria* | 0.98 | 0.91 | 0.97 | 0.89 | 0.92 | 0.74 | 0.96 | 0.91 | 0.98 | 0.92 |
| *Ricinodendron heudelotii* | 0.98 | 0.93 | 0.97 | 0.89 | 0.90 | 0.68 | 0.91 | 0.83 | 0.97 | 0.86 |
| *Spondias mombin* | 0.92 | 0.74 | 0.90 | 0.72 | 0.85 | 0.61 | 0.89 | 0.75 | 0.92 | 0.76 |
| *Tectona grandis* | 0.97 | 0.82 | 0.96 | 0.82 | 0.92 | 0.69 | 0.92 | 0.82 | 0.97 | 0.82 |
| *Terminalia ivorensis* | 0.93 | 0.82 | 0.82 | 0.64 | 0.92 | 0.75 | 0.90 | 0.68 | 0.94 | 0.84 |
| *Terminalia superba* | 0.99 | 0.92 | 0.90 | 0.81 | 0.92 | 0.77 | 0.96 | 0.91 | 0.99 | 0.95 |
| *Tetrapleura tetraptera* | 0.98 | 0.87 | 0.87 | 0.75 | 0.91 | 0.74 | 0.96 | 0.91 | 0.97 | 0.91 |
| *Theobroma cacao* | 0.96 | 0.80 | 0.94 | 0.80 | 0.92 | 0.77 | 0.93 | 0.83 | 0.96 | 0.84 |
| *Triplochiton scleroxylon* | 1.00 | 0.94 | 0.96 | 0.92 | 0.97 | 0.87 | 0.99 | 0.95 | 1.00 | 0.96 |

Table S4. Relative importance values (%) for environmental predictors by species. Variable description in Table 1.

| specie | BIO2 | BIO4 | BIO8 | BIO9 | BIO13 | BIO15 | BIO18 | BIO19 | phh2o | cec | nitrogen | sand |
| --- | --- | --- | --- | --- | --- | --- | --- | --- | --- | --- | --- | --- |
| *Acacia mangium* | 25.46 | 8.77 | 5.06 | 6.04 | 25.13 | 1.60 | 6.06 | 4.86 | 9.65 | 2.87 | 2.33 | 2.17 |
| *Albizia guachapele* | 4.13 | 56.14 | 1.46 | 1.39 | 2.67 | 2.10 | 2.08 | 1.36 | 9.31 | 5.88 | 10.34 | 3.13 |
| *Albizia lebbeck* | 2.84 | 36.00 | 10.54 | 4.45 | 8.29 | 2.13 | 15.01 | 5.52 | 6.85 | 3.52 | 2.20 | 2.65 |
| *Alstonia boonei* | 6.51 | 64.02 | 0.85 | 1.16 | 4.48 | 4.34 | 5.20 | 8.03 | 0.63 | 0.77 | 0.94 | 3.08 |
| *Anacardium occidentale* | 7.66 | 63.05 | 2.83 | 3.35 | 3.18 | 2.65 | 1.75 | 5.02 | 6.12 | 1.17 | 1.59 | 1.63 |
| *Artocarpus altilis* | 4.62 | 46.06 | 1.58 | 3.59 | 4.09 | 3.25 | 8.24 | 14.01 | 3.34 | 5.20 | 4.60 | 1.42 |
| *Carica papaya* | 3.81 | 38.27 | 3.31 | 4.36 | 7.78 | 2.29 | 9.97 | 7.91 | 5.62 | 10.06 | 5.42 | 1.21 |
| *Ceiba pentandra* | 4.95 | 42.90 | 2.35 | 11.27 | 11.47 | 2.80 | 1.65 | 6.11 | 7.45 | 2.53 | 4.76 | 1.75 |
| *Citrus grandis* | 3.54 | 4.04 | 2.61 | 2.85 | 12.04 | 2.70 | 38.28 | 3.95 | 3.46 | 4.28 | 15.11 | 7.15 |
| *Citrus reticulata* | 5.70 | 12.42 | 2.80 | 9.61 | 8.16 | 1.95 | 14.33 | 14.13 | 5.90 | 4.14 | 18.78 | 2.09 |
| *Cocos nucifera* | 7.18 | 28.98 | 10.42 | 6.30 | 15.09 | 2.01 | 5.73 | 7.32 | 6.35 | 3.17 | 5.53 | 1.93 |
| *Cola acuminata* | 39.13 | 35.02 | 1.04 | 2.36 | 2.00 | 4.93 | 4.53 | 7.61 | 0.77 | 1.14 | 0.46 | 1.02 |
| *Cola nitida* | 4.48 | 60.16 | 0.61 | 1.93 | 1.97 | 5.87 | 2.10 | 20.39 | 0.57 | 0.48 | 0.97 | 0.46 |
| *Elaeis guineensis* | 2.04 | 49.36 | 0.88 | 7.13 | 6.18 | 3.93 | 3.23 | 17.72 | 4.52 | 1.03 | 2.16 | 1.84 |
| *Entandrophragma angolense* | 1.72 | 66.39 | 1.11 | 3.42 | 2.21 | 6.14 | 6.16 | 1.95 | 2.78 | 1.08 | 1.69 | 5.35 |
| *Funtumia elastica* | 3.74 | 35.73 | 1.49 | 1.66 | 1.57 | 3.06 | 5.49 | 39.63 | 0.83 | 1.43 | 0.60 | 4.78 |
| *Garcinia kola* | 1.28 | 75.53 | 0.36 | 0.55 | 1.93 | 9.60 | 3.24 | 0.91 | 0.46 | 0.79 | 0.43 | 4.91 |
| *Gliricidia sepium* | 1.92 | 48.75 | 1.49 | 3.00 | 4.67 | 2.90 | 6.00 | 2.09 | 4.70 | 20.12 | 3.93 | 0.42 |
| *Gmelina arborea* | 3.98 | 18.32 | 4.14 | 8.18 | 29.22 | 5.31 | 4.90 | 2.04 | 15.96 | 4.42 | 2.03 | 1.49 |
| *Hevea brasiliensis* | 2.96 | 47.37 | 3.73 | 3.94 | 9.08 | 6.78 | 7.25 | 6.30 | 8.10 | 1.42 | 1.58 | 1.49 |
| *Irvingia gabonensis* | 7.99 | 77.06 | 0.33 | 1.69 | 0.23 | 7.32 | 0.94 | 1.04 | 0.43 | 0.50 | 0.41 | 2.04 |
| *Lophira alata* | 3.26 | 63.38 | 0.47 | 1.25 | 4.26 | 4.14 | 7.31 | 2.37 | 8.47 | 0.50 | 0.85 | 3.74 |
| *Mangifera indica* | 3.34 | 46.74 | 1.83 | 3.69 | 10.80 | 1.93 | 4.59 | 6.56 | 10.00 | 4.26 | 2.90 | 3.36 |
| *Milicia excelsa* | 1.98 | 56.72 | 2.48 | 2.49 | 3.22 | 3.05 | 2.12 | 16.83 | 0.85 | 1.09 | 0.74 | 8.43 |
| *Musa paradisiaca* | 3.63 | 27.90 | 4.39 | 8.57 | 13.60 | 3.62 | 4.27 | 9.12 | 5.72 | 9.30 | 6.53 | 3.36 |
| *Pachylobus edulis* | 6.53 | 54.94 | 3.36 | 0.87 | 1.72 | 1.23 | 0.84 | 0.85 | 20.22 | 2.27 | 1.92 | 5.26 |
| *Persea americana* | 2.79 | 40.28 | 3.48 | 5.03 | 1.97 | 1.53 | 4.80 | 6.40 | 3.18 | 4.13 | 25.11 | 1.29 |
| *Psidium guajava* | 4.72 | 29.90 | 3.65 | 5.96 | 19.63 | 1.44 | 6.96 | 8.62 | 2.53 | 3.91 | 11.33 | 1.34 |
| *Pycnanthus angolensis* | 1.10 | 67.65 | 2.26 | 2.87 | 2.13 | 6.30 | 3.38 | 5.55 | 1.49 | 1.06 | 0.76 | 5.47 |
| *Rauvolfia vomitoria* | 4.05 | 68.15 | 1.62 | 1.34 | 5.78 | 5.21 | 3.43 | 6.43 | 1.08 | 1.27 | 0.93 | 0.71 |
| *Ricinodendron heudelotii* | 4.72 | 65.63 | 3.76 | 2.57 | 6.90 | 1.95 | 2.51 | 3.67 | 1.65 | 1.29 | 0.84 | 4.50 |
| *Spondias mombin* | 1.71 | 59.03 | 5.49 | 2.95 | 5.94 | 2.65 | 2.66 | 6.48 | 5.17 | 2.98 | 2.90 | 2.03 |
| *Tectona grandis* | 1.49 | 47.20 | 1.61 | 19.42 | 5.11 | 1.12 | 2.21 | 1.70 | 13.98 | 1.90 | 2.86 | 1.40 |
| *Terminalia ivorensis* | 1.28 | 64.21 | 1.23 | 4.33 | 4.83 | 1.89 | 3.05 | 10.84 | 1.24 | 2.83 | 0.84 | 3.41 |
| *Terminalia superba* | 2.55 | 27.76 | 1.47 | 15.78 | 3.47 | 6.25 | 1.35 | 34.35 | 3.76 | 0.67 | 0.47 | 2.12 |
| *Tetrapleura tetraptera* | 3.19 | 67.89 | 2.65 | 5.10 | 2.57 | 1.86 | 2.77 | 4.32 | 2.80 | 0.84 | 3.13 | 2.89 |
| *Theobroma cacao* | 3.30 | 52.28 | 3.05 | 3.21 | 2.82 | 2.84 | 12.65 | 6.99 | 2.70 | 3.98 | 4.03 | 2.14 |
| *Triplochiton scleroxylon* | 1.31 | 54.31 | 1.15 | 4.88 | 2.20 | 5.78 | 0.90 | 25.51 | 1.61 | 0.28 | 0.67 | 1.40 |

Table S5. Projected changes of key bioclimate variables between current and 2021-2040 climates in West Africa, considering two Shared Socio-economic Pathways. CanESM5 is the more climate sensitivity model of the CMIP6 models (see more in https://www.carbonbrief.org/cmip6-the-next-generation-of-climate-models-explained) and MIROC6 is the lowest sensitivity. Variable description in Table 1.

| **Variable** | **Current** | | | **2021 - 2040** | | | | | | | | | | | | | | | | | |
| --- | --- | --- | --- | --- | --- | --- | --- | --- | --- | --- | --- | --- | --- | --- | --- | --- | --- | --- | --- | --- | --- |
|  |  |  |  | **CanESM5** | | | | | | **Averaged models** | | | | | | **MIROC6** | | | | | |
|  |  |  |  | **SSP 126** | | | **SSP 585** | | | **SSP 126** | | | **SSP 585** | | | **SSP 126** | | | **SSP 585** | | |
|  | **Av** | **Max** | **Min** | **Av** | **Max** | **Min** | **Av** | **Max** | **Min** | **Av** | **Max** | **Min** | **Av** | **Max** | **Min** | **Av** | **Max** | **Min** | **Av** | **Max** | **Min** |
| BIO2 | 11.42 | 17.20 | 5.47 | 11.10 | 17.11 | 5.44 | 10.82 | 16.91 | 5.38 | 11.26 | 17.43 | 5.24 | 11.17 | 17.38 | 5.31 | 11.46 | 17.43 | 5.61 | 11.42 | 17.38 | 5.64 |
| BIO4 | 164.61 | 372.86 | 51.13 | 177.41 | 346.02 | 54.02 | 181.73 | 344.16 | 55.16 | 174.24 | 377.62 | 45.24 | 174.35 | 390.07 | 43.32 | 172.56 | 377.51 | 50.91 | 170.68 | 363.51 | 49.47 |
| BIO8 | 25.20 | 28.87 | 9.17 | 26.44 | 30.30 | 10.47 | 26.54 | 30.17 | 10.65 | 26.29 | 30.30 | 10.05 | 26.33 | 30.17 | 10.13 | 25.91 | 29.15 | 10.10 | 26.01 | 29.20 | 10.18 |
| BIO9 | 25.67 | 30.19 | 11.76 | 27.60 | 32.07 | 13.40 | 27.73 | 32.27 | 13.68 | 27.04 | 32.07 | 12.48 | 26.97 | 32.27 | 12.43 | 26.69 | 31.07 | 12.65 | 26.72 | 31.17 | 12.63 |
| BIO13 | 290.30 | 1115 | 113 | 350.23 | 1286 | 158 | 372.47 | 1257 | 165 | 312.15 | 1343 | 130 | 324.22 | 1275 | 134 | 320.51 | 1162 | 151 | 307.48 | 1117 | 145 |
| BIO15 | 92.29 | 158.67 | 36.06 | 94.95 | 165.99 | 37.74 | 95.57 | 166.03 | 38.27 | 93.80 | 171.41 | 31.22 | 94.53 | 180.13 | 33.96 | 95.18 | 171.41 | 34.43 | 93.53 | 163.20 | 33.96 |
| BIO18 | 211.5 | 844 | 12 | 210.13 | 861 | 6 | 211.43 | 961 | 4 | 205.18 | 934 | 6 | 210.79 | 972 | 4 | 210.40 | 811 | 9 | 206.18 | 924 | 9 |
| BIO19 | 459.7 | 2728 | 0 | 633.08 | 3116 | 0 | 638.82 | 3090 | 0 | 518.97 | 3146 | 0 | 512.84 | 3161 | 0 | 526.09 | 2815 | 0 | 506.93 | 2721 | 0 |

Table S6. Projected changes of key bioclimate variables between current and 2041-2060 climates in West Africa, considering two Shared Socio-economic Pathways. CanESM5 is the more climate sensitivity model of the CMIP6 models (see more in https://www.carbonbrief.org/cmip6-the-next-generation-of-climate-models-explained) and MIROC6 is the lowest sensitivity. Variable description in Table 1.

| **Variable** | **Current** | | | **2041 - 2060** | | | | | | | | | | | | | | | | | |
| --- | --- | --- | --- | --- | --- | --- | --- | --- | --- | --- | --- | --- | --- | --- | --- | --- | --- | --- | --- | --- | --- |
|  |  |  |  | **CanESM5** | | | | | | **Averaged models** | | | | | | **MIROC6** | | | | | |
|  |  |  |  | **SSP 126** | | | **SSP 585** | | | **SSP 126** | | | **SSP 585** | | | **SSP 126** | | | **SSP 585** | | |
|  | **Av** | **Max** | **Min** | **Av** | **Max** | **Min** | **Av** | **Max** | **Min** | **Av** | **Max** | **Min** | **Av** | **Max** | **Min** | **Av** | **Max** | **Min** | **Av** | **Max** | **Min** |
| BIO2 | 11.42 | 17.20 | 5.47 | 11.05 | 17.07 | 5.42 | 10.78 | 17.10 | 5.52 | 11.26 | 17.51 | 5.39 | 11.14 | 17.97 | 5.24 | 11.51 | 17.51 | 5.61 | 11.76 | 17.97 | 5.77 |
| BIO4 | 164.61 | 372.86 | 51.13 | 181.78 | 348.51 | 54.79 | 192.48 | 354.79 | 57.24 | 174.41 | 391.70 | 44.79 | 183.03 | 396.99 | 45.94 | 174.69 | 379.65 | 50.11 | 187.69 | 396.99 | 49.69 |
| BIO8 | 25.20 | 28.87 | 9.17 | 26.81 | 30.73 | 10.80 | 27.71 | 31.23 | 11.78 | 26.64 | 30.73 | 10.33 | 27.33 | 31.23 | 10.85 | 26.13 | 29.28 | 10.35 | 26.70 | 29.85 | 11.02 |
| BIO9 | 25.67 | 30.19 | 11.76 | 28.10 | 32.46 | 13.85 | 29.26 | 33.60 | 14.98 | 27.36 | 32.47 | 12.67 | 28.11 | 33.60 | 13.20 | 26.94 | 31.47 | 12.90 | 27.62 | 31.88 | 13.78 |
| BIO13 | 290.30 | 1115 | 113 | 360.27 | 1288 | 157 | 397.74 | 1317 | 164 | 318.12 | 1294 | 134 | 325.28 | 1317 | 125 | 319.80 | 1172 | 149 | 315.28 | 1171 | 151 |
| BIO15 | 92.29 | 158.67 | 36.06 | 95.21 | 167.12 | 38.30 | 96.43 | 169 | 39.22 | 94.55 | 173.66 | 37.22 | 94.68 | 177.99 | 37.95 | 95.24 | 173.266 | 37.22 | 95.96 | 171.82 | 37.95 |
| BIO18 | 211.5 | 844 | 12 | 211.42 | 742 | 6 | 199.52 | 959 | 3 | 208.74 | 938 | 6 | 200 | 1033 | 3 | 209.37 | 760 | 11 | 194.78 | 727 | 6 |
| BIO19 | 459.7 | 2728 | 0 | 668.63 | 3158 | 0 | 719.23 | 3218 | 0 | 529.55 | 3279 | 0 | 529.27 | 3333 | 0 | 525.30 | 2814 | 0 | 536.92 | 2714 | 0 |

**FIGURES**

**
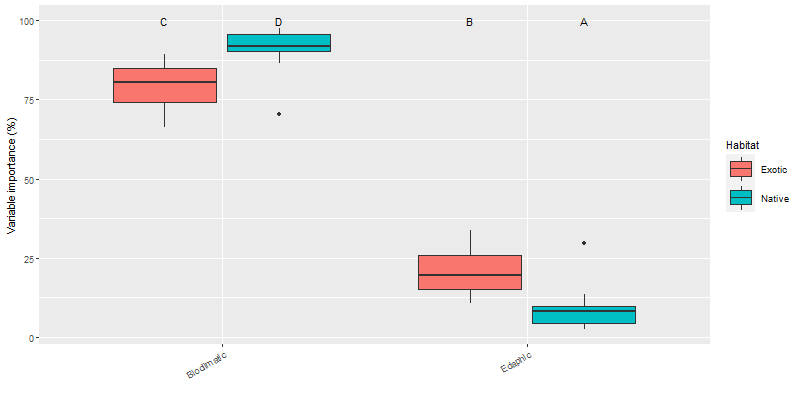
**

Figure S1. Accumulated importance of bioclimatic and edaphic variables (%) per shade tree species by habitat status (i.e. exotic and native) in West Africa considering the GBM algorithm. Letters above indicate significant differences between levels (*p* <0.05) according to Tukey post-hoc test.

**
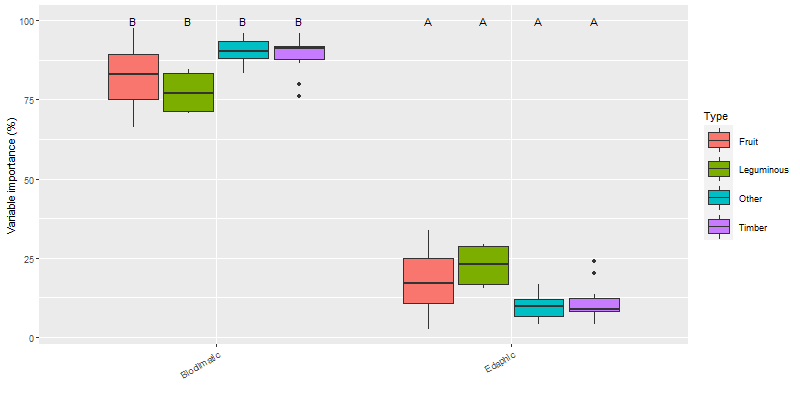
**

Figure S2. Accumulated importance of bioclimatic and edaphic variables (%) per shade tree species by main use in West Africa considering the GBM algorithm. Letters above indicate significant differences between levels (*p* <0.05) according to Tukey post-hoc test.

**
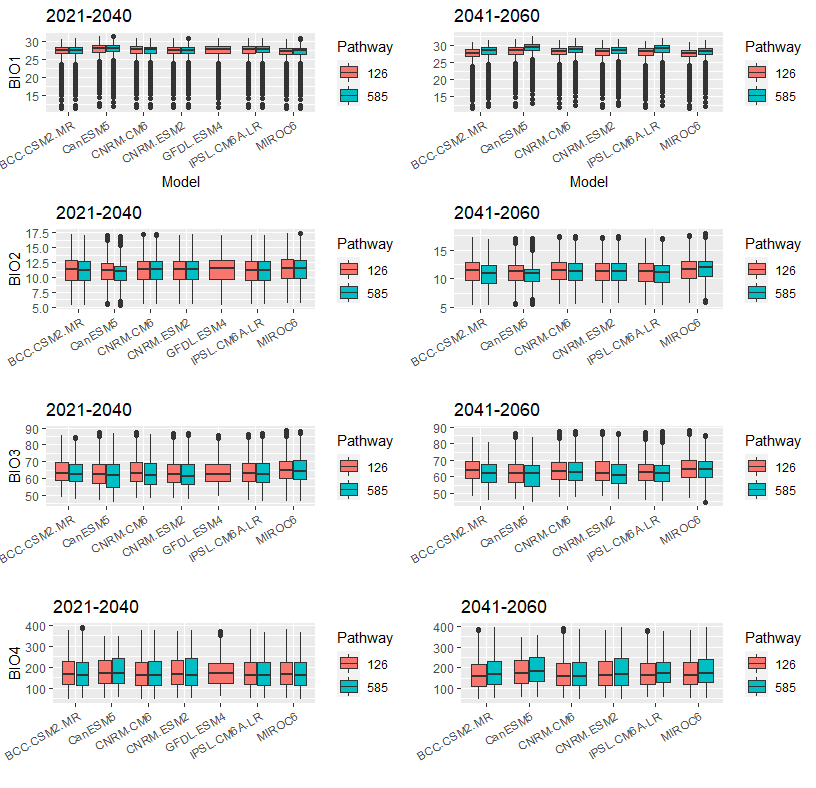
**

Figure S3. Boxplots of the bioclimatic variables (BIO1:BIO4) under projected climate conditions by different CMIP6 models (<https://www.carbonbrief.org/cmip6-the-next-generation-of-climate-models-explained>) and classified according to the Shared Socio-economic Pathways. Boxes represents upper and lower quartiles and median is drawn as a horizontal solid line. The full names and meanings of each variable can be found in Table 1 and <https://worldclim.org/>

**
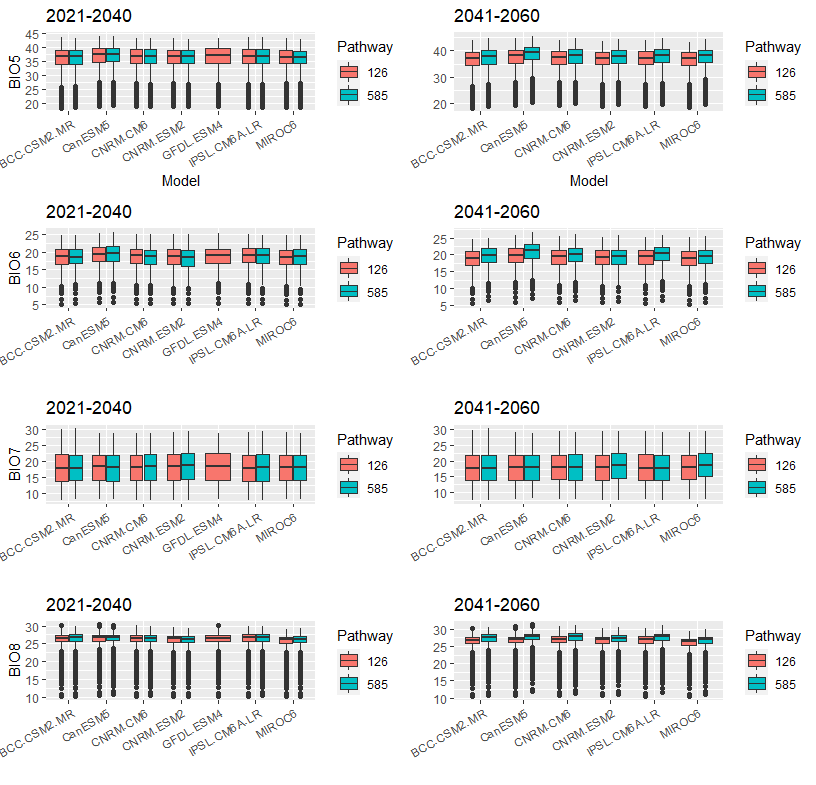
**

Figure S4. Boxplots of the bioclimatic variables (BIO5:BIO8) under projected climate conditions by different CMIP6 models (<https://www.carbonbrief.org/cmip6-the-next-generation-of-climate-models-explained>) and classified according to the Shared Socio-economic Pathways. Boxes represents upper and lower quartiles and median is drawn as a horizontal solid line. The full names and meanings of each variable can be found in Table 1 and <https://worldclim.org/>

**
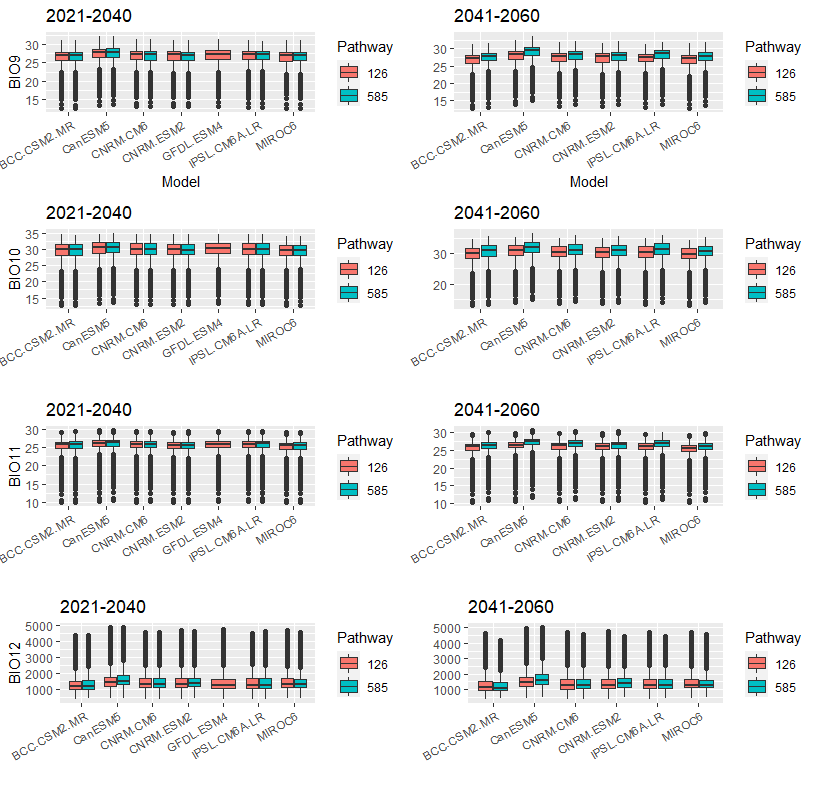
**

Figure S5. Boxplots of the bioclimatic variables (BIO9:BIO12) under projected climate conditions by different CMIP6 models (<https://www.carbonbrief.org/cmip6-the-next-generation-of-climate-models-explained>) and classified according to the Shared Socio-economic Pathways. Boxes represents upper and lower quartiles and median is drawn as a horizontal solid line. The full names and meanings of each variable can be found in Table 1 and <https://worldclim.org/>

**
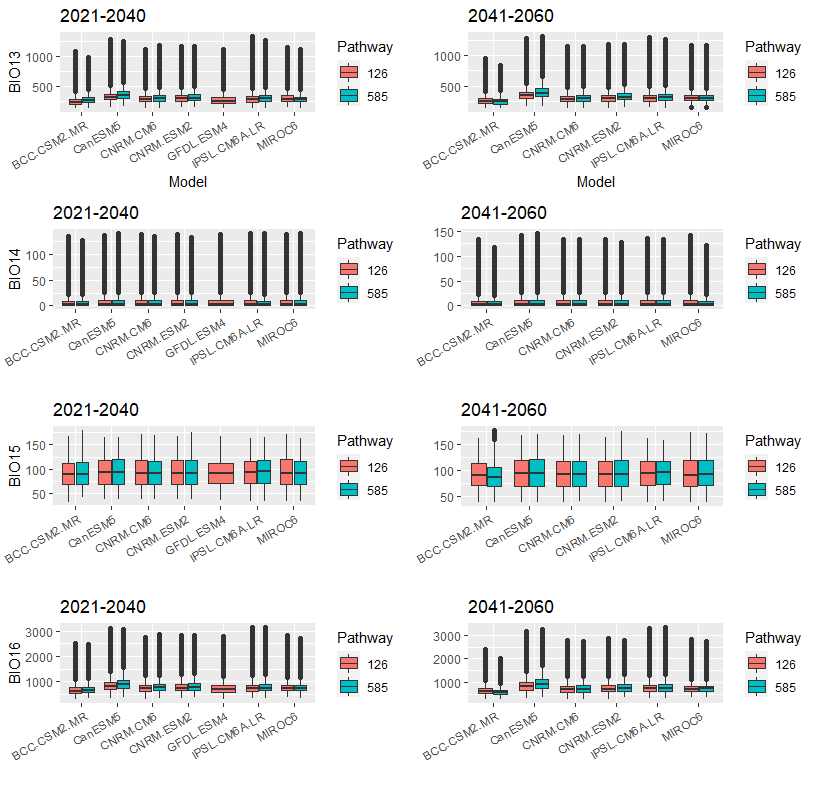
**

Figure S6. Boxplots of the bioclimatic variables (BIO13:BIO16) under projected climate conditions by different CMIP6 models (<https://www.carbonbrief.org/cmip6-the-next-generation-of-climate-models-explained>) and classified according to the Shared Socio-economic Pathways. Boxes represents upper and lower quartiles and median is drawn as a horizontal solid line. The full names and meanings of each variable can be found in Table 1 and <https://worldclim.org/>

**
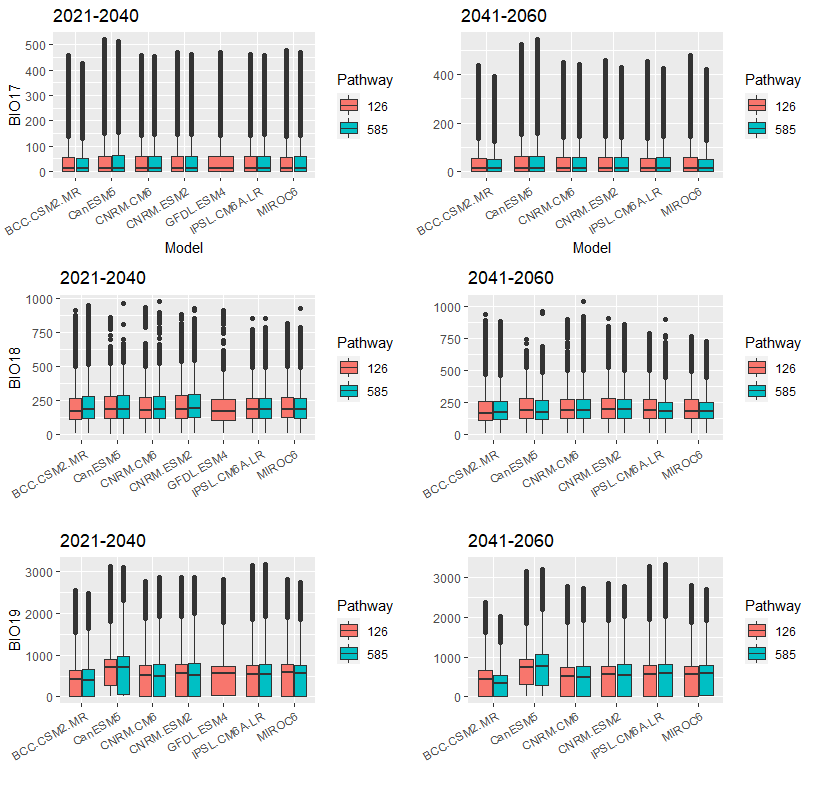
**

Figure S7. Boxplots of the bioclimatic variables (BIO17:BIO19) under projected climate conditions by different CMIP6 models (<https://www.carbonbrief.org/cmip6-the-next-generation-of-climate-models-explained>) and classified according to the Shared Socio-economic Pathways. Boxes represents upper and lower quartiles and median is drawn as a horizontal solid line. The full names and meanings of each variable can be found in Table 1 and <https://worldclim.org/>

**
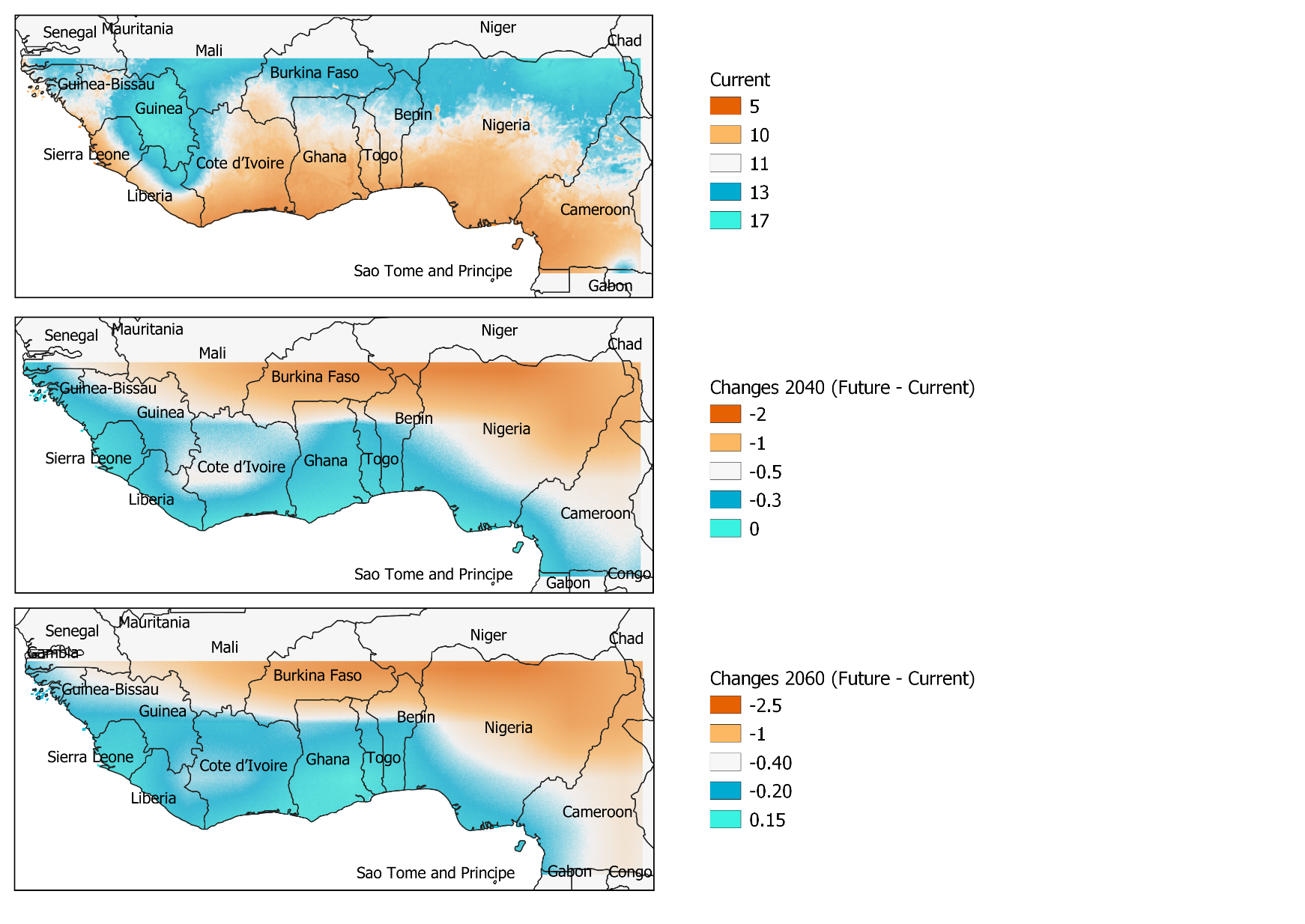
**Figure S8. Mean Diurnal Range (ºC) under current conditions, as well as shifts due to climate change by 2021-2040 and 2041-2060 in West Africa, considering the high emission Shared Socio-economic Pathway: 585. Variable description in Table 1 and <https://worldclim.org/>*.*

Graphs were generated by QGIS 3.26.3 (<https://www.qgis.org>) with the global vector data from the GADM database (<https://gadm.org>).

**
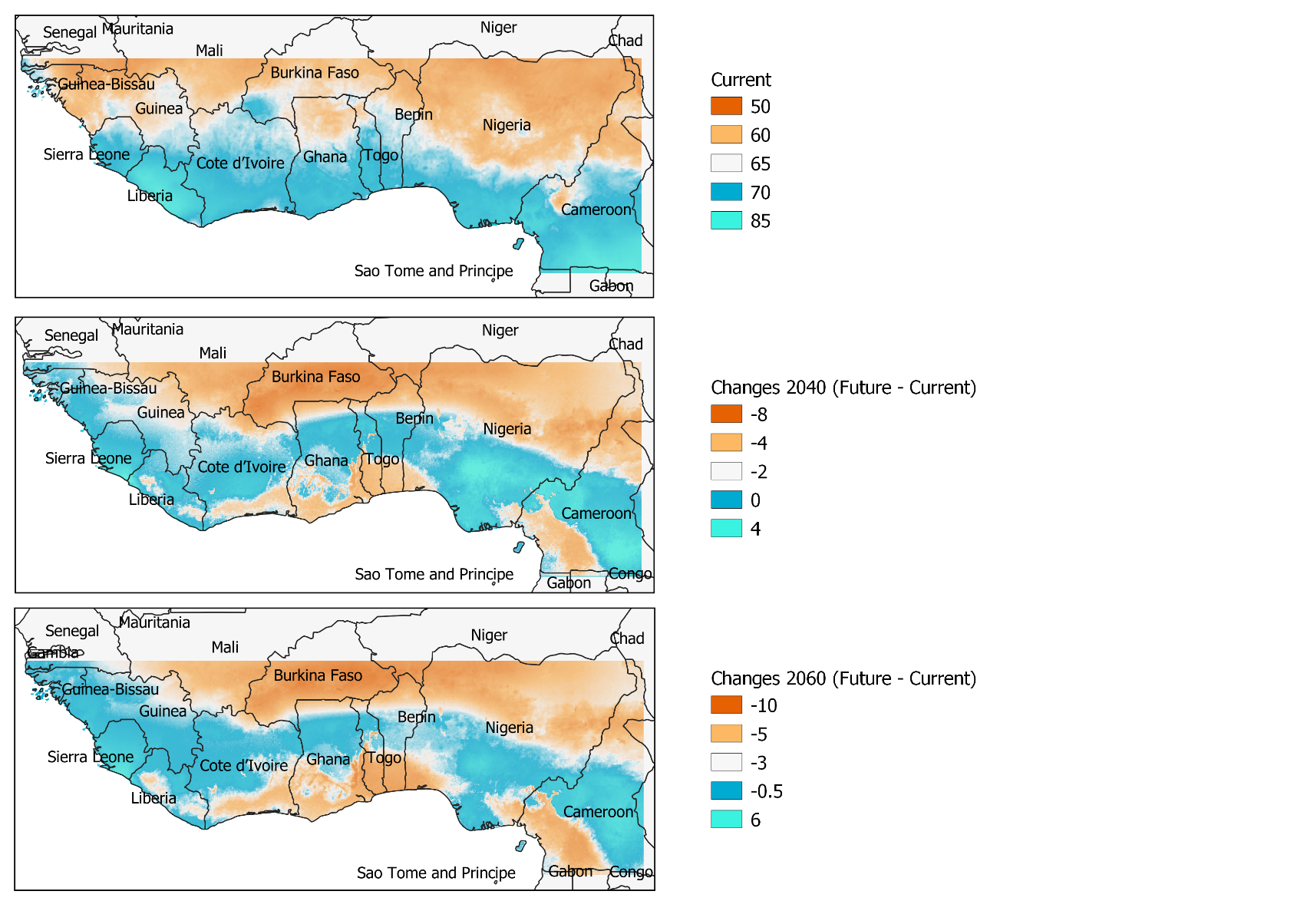
**

Figure S9. Isothermality under current conditions, as well as shifts due to climate change by 2021-2040 and 2041-2060 in West Africa, considering the high emission Shared Socio-economic Pathway: 585. Variable description in Table 1 and <https://worldclim.org/>

Graphs were generated by QGIS 3.26.3 (<https://www.qgis.org>) with the global vector data from the GADM database (<https://gadm.org>).

**
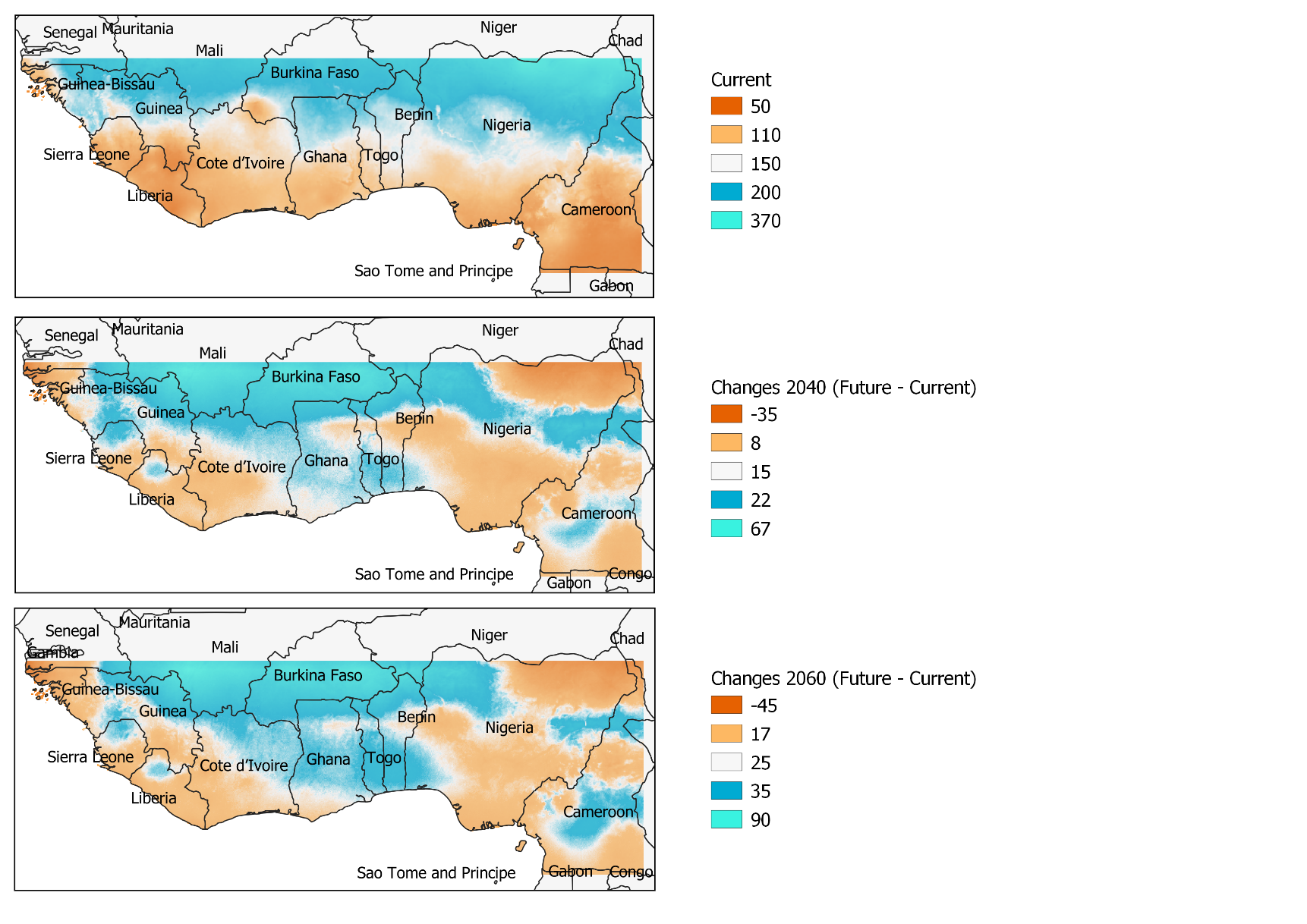
**

Figure S10. Temperature Seasonality under current conditions, as well as shifts due to climate change by 2021-2040 and 2041-2060 in West Africa, considering the high emission Shared Socio-economic Pathway: 585. Variable description in Table 1 and <https://worldclim.org/>

Graphs were generated by QGIS 3.26.3 (<https://www.qgis.org>) with the global vector data from the GADM database (<https://gadm.org>).

**
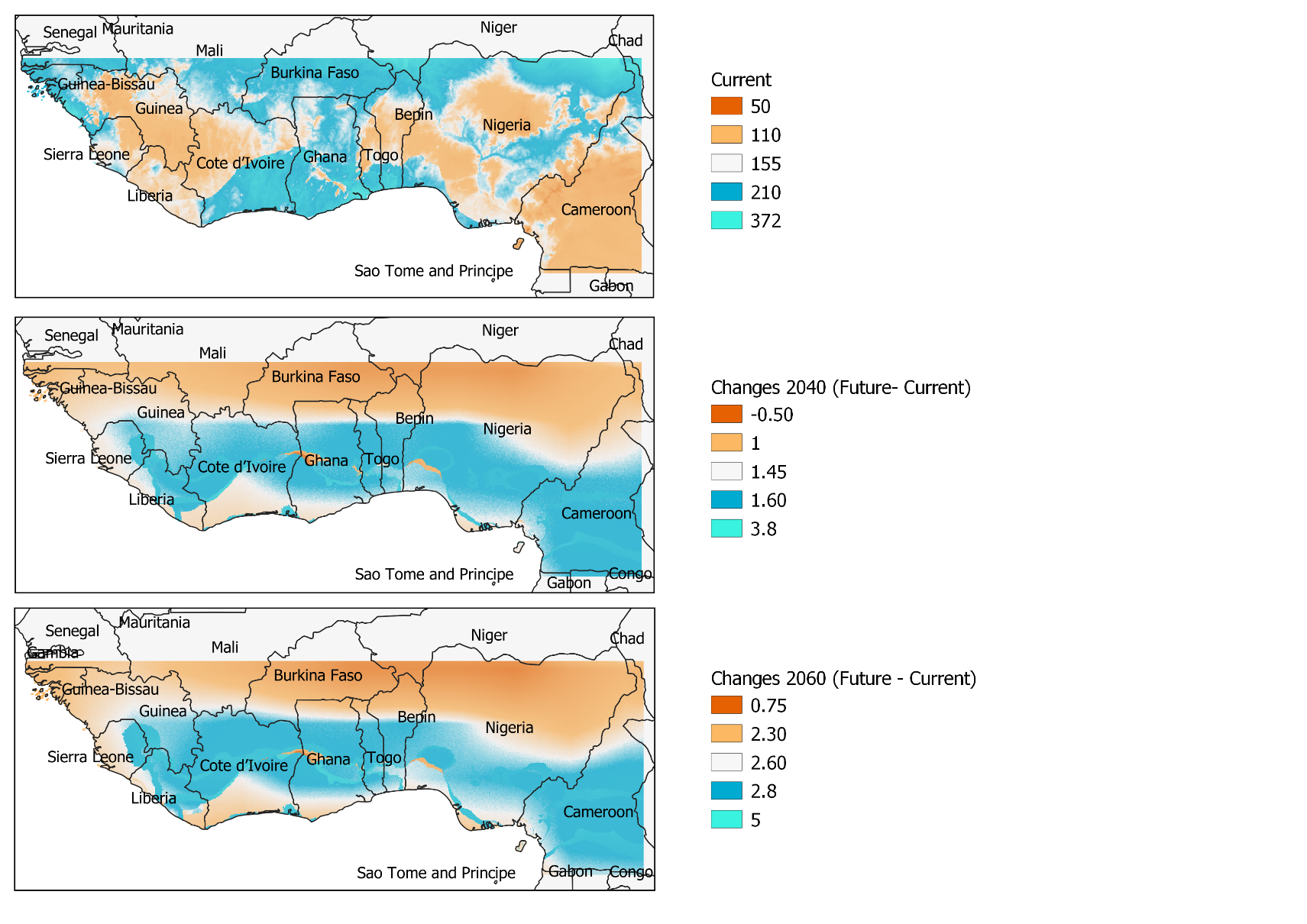
**

Figure S11. Mean Temperature of Wettest Quarter (ºC) under current conditions, as well as shifts due to climate change by 2021-2040 and 2041-2060 in West Africa, considering the high emission Shared Socio-economic Pathway: 585. Variable description in Table 1 and <https://worldclim.org/>

Graphs were generated by QGIS 3.26.3 (<https://www.qgis.org>) with the global vector data from the GADM database (<https://gadm.org>).

**
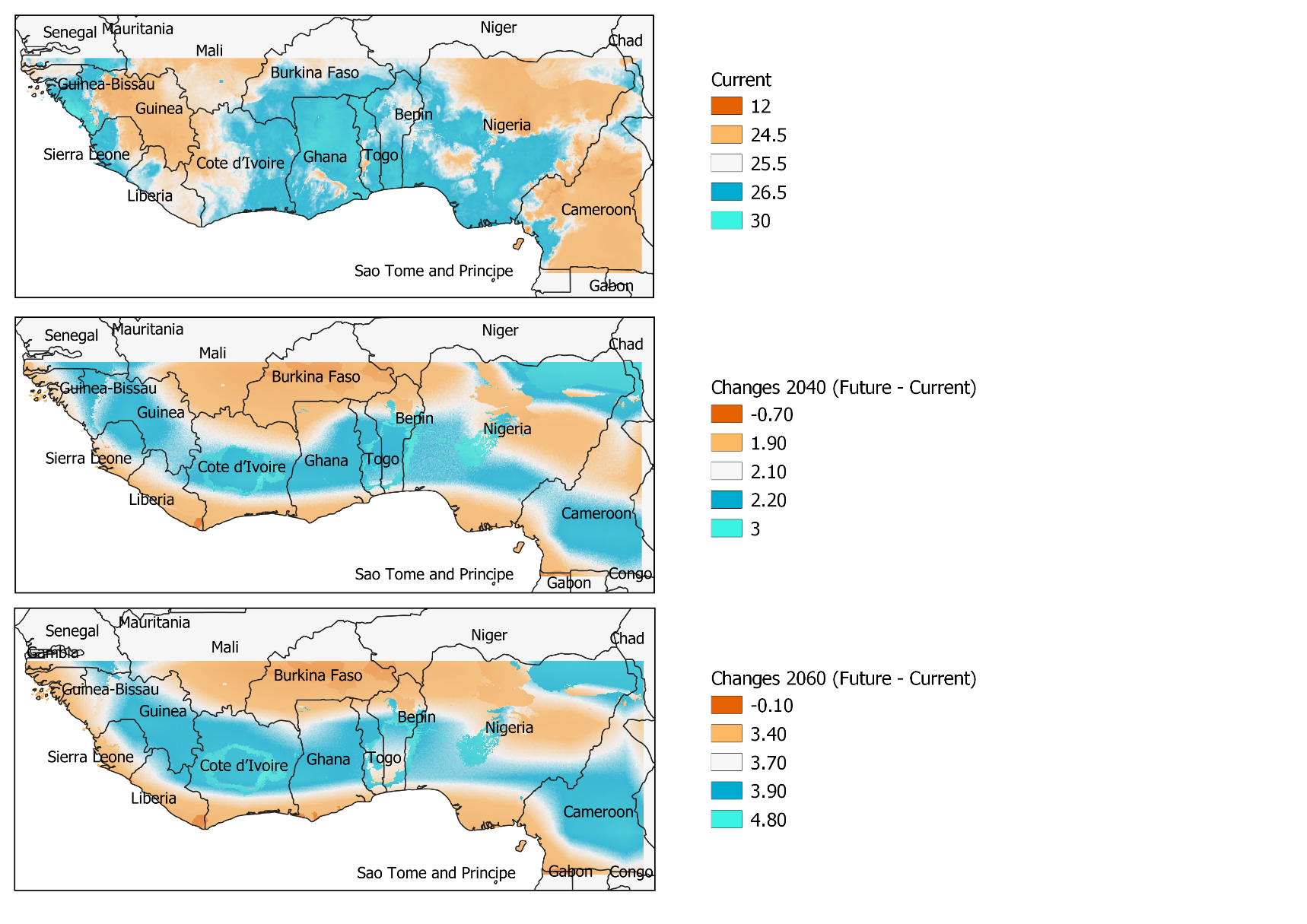
**

Figure S12. Mean Temperature of Driest Quarter (ºC) under current conditions, as well as shifts due to climate change by 2021-2040 and 2041-2060 in West Africa, considering the high emission Shared Socio-economic Pathway: 585. Variable description in Table 1 and <https://worldclim.org/>

Graphs were generated by QGIS 3.26.3 (<https://www.qgis.org>) with the global vector data from the GADM database (<https://gadm.org>).

**
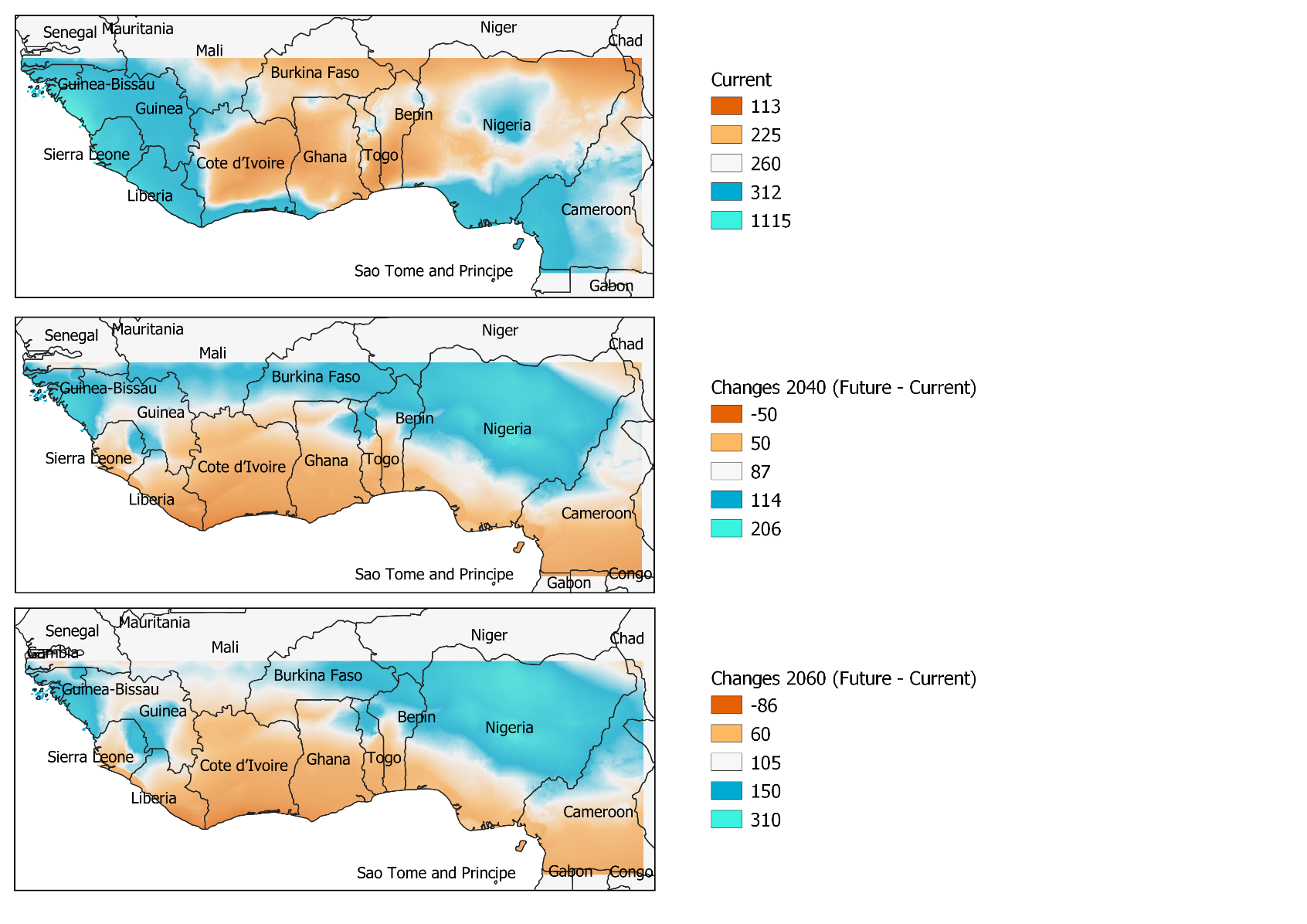
**

Figure S13. Precipitation of Wettest Month (mm) under current conditions, as well as shifts due to climate change by 2021-2040 and 2041-2060 in West Africa, considering the high emission Shared Socio-economic Pathway: 585. Variable description in Table 1 and <https://worldclim.org/>

Graphs were generated by QGIS 3.26.3 (<https://www.qgis.org>) with the global vector data from the GADM database (<https://gadm.org>).

**
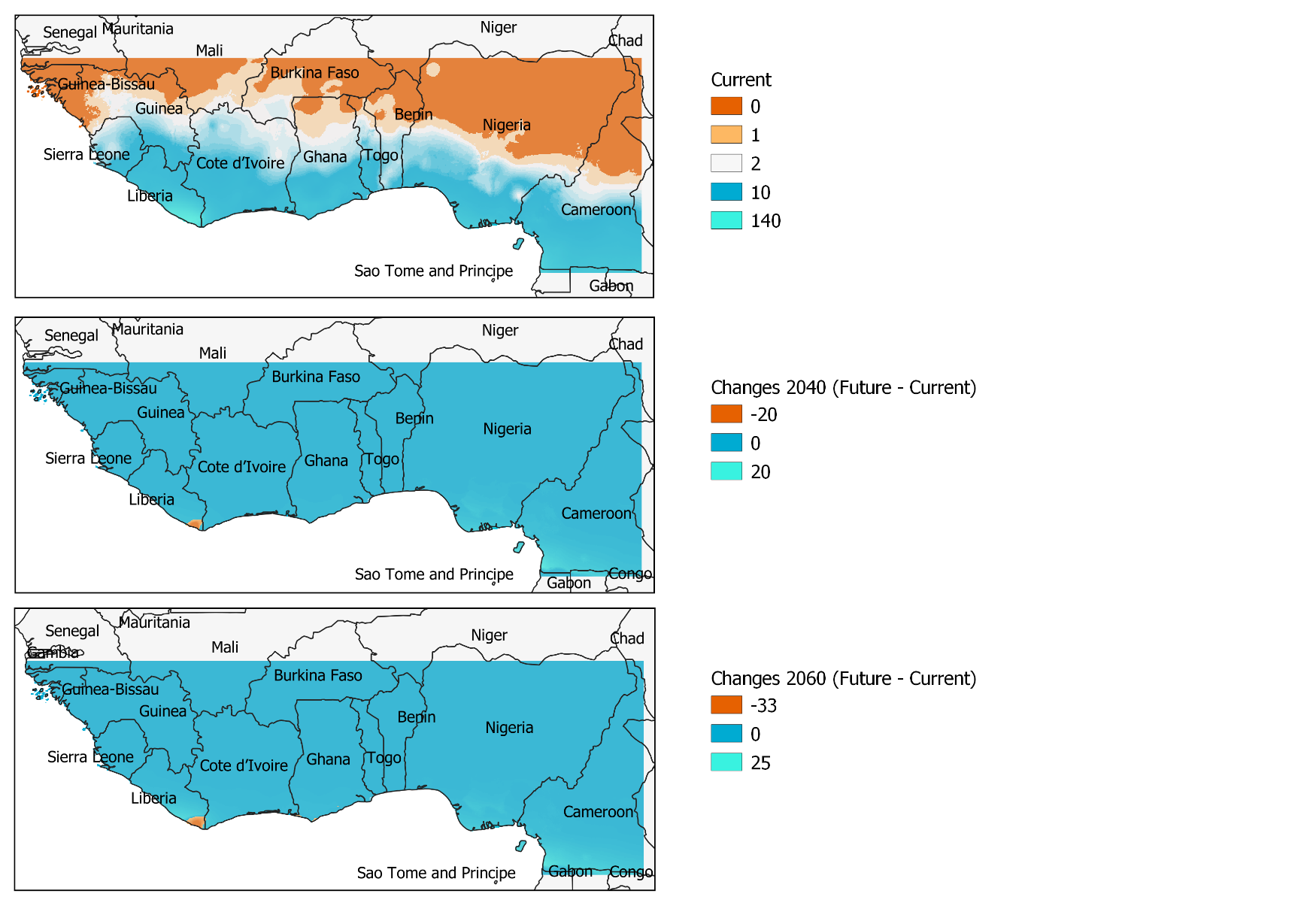
**

Figure S14. Precipitation of Driest Month (mm) under current conditions, as well as shifts due to climate change by 2021-2040 and 2041-2060 in West Africa, considering the high emission Shared Socio-economic Pathway: 585. Variable description in Table 1 and <https://worldclim.org/>

Graphs were generated by QGIS 3.26.3 (<https://www.qgis.org>) with the global vector data from the GADM database (<https://gadm.org>).

**
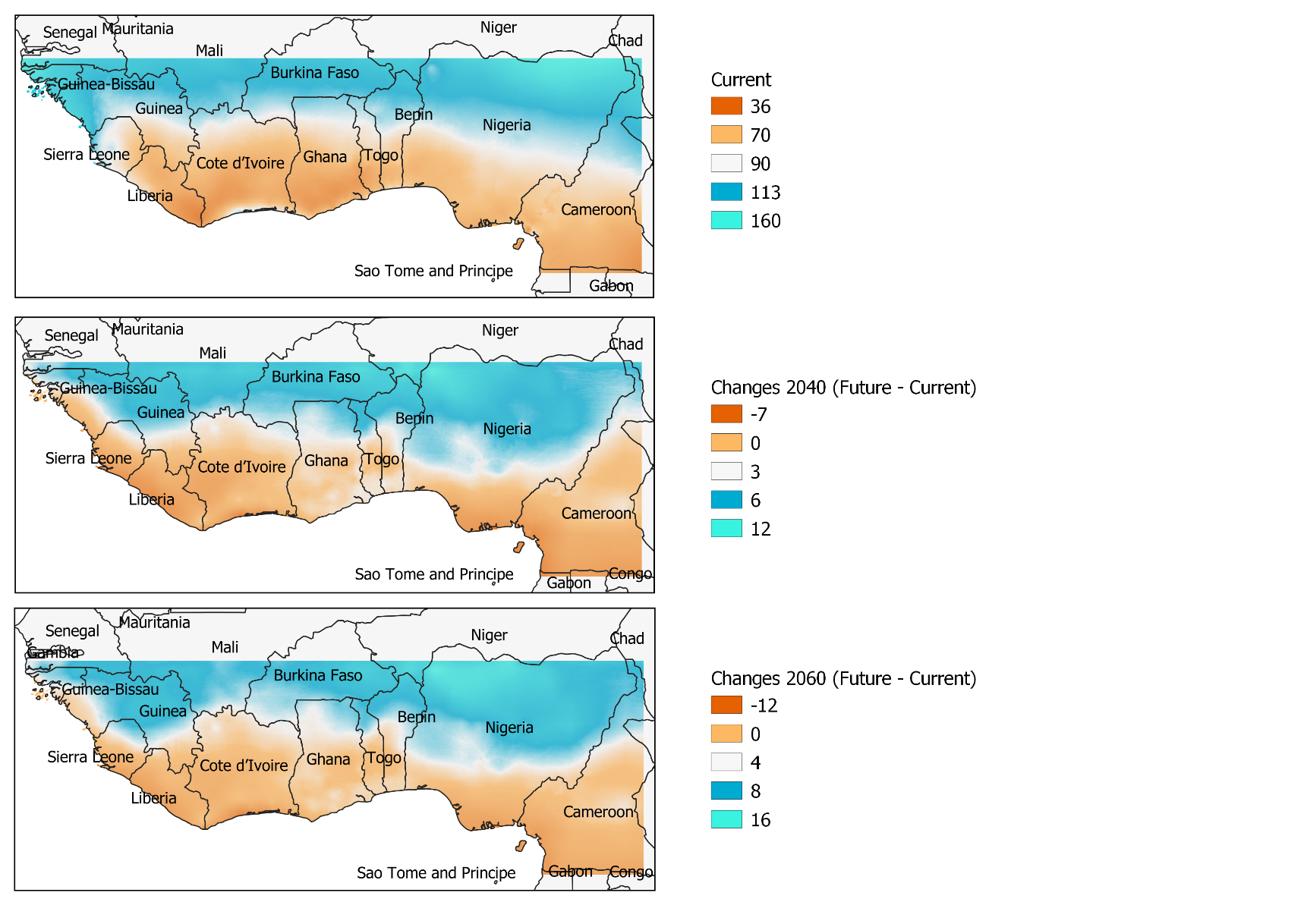
**

Figure S15. Precipitation Seasonality under current conditions, as well as shifts due to climate change by 2021-2040 and 2041-2060 in West Africa, considering the high emission Shared Socio-economic Pathway: 585. Variable description in Table 1 and <https://worldclim.org/>

Graphs were generated by QGIS 3.26.3 (<https://www.qgis.org>) with the global vector data from the GADM database (<https://gadm.org>).

**
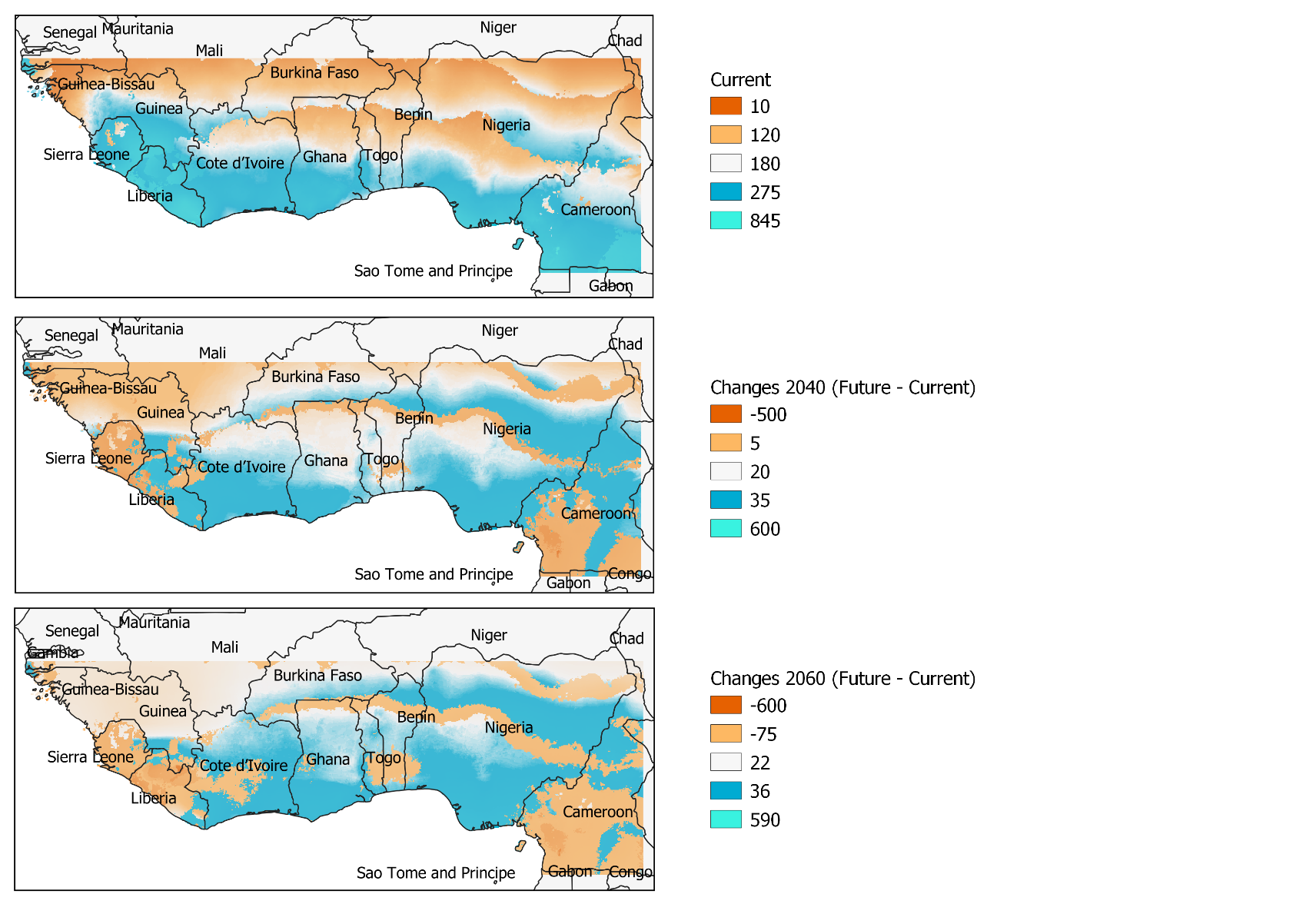
**

Figure S16. Precipitation of Warmest Quarter (mm) under current conditions, as well as shifts due to climate change by 2021-2040 and 2041-2060 in West Africa, considering the high emission Shared Socio-economic Pathway: 585. Variable description in Table 1 and <https://worldclim.org/>

Graphs were generated by QGIS 3.26.3 (<https://www.qgis.org>) with the global vector data from the GADM database (<https://gadm.org>).

**
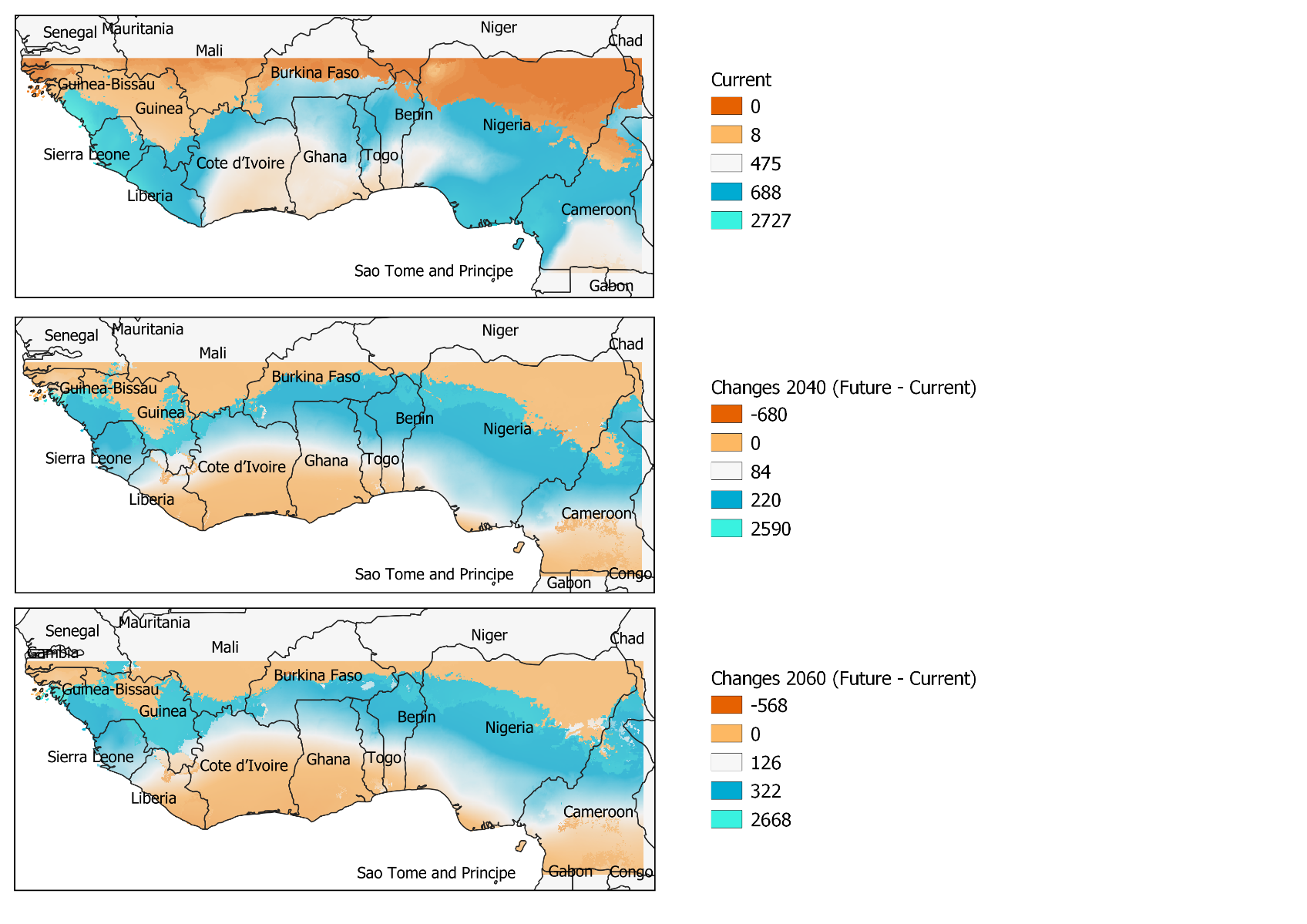
**

Figure S17. Precipitation of Coldest Quarter (mm) under current conditions, as well as shifts due to climate change by 2021-2040 and 2041-2060 in West Africa, considering the high emission Shared Socio-economic Pathway: 585. Variable description in Table 1 and <https://worldclim.org/>

Graphs were generated by QGIS 3.26.3 (<https://www.qgis.org>) with the global vector data from the GADM database (<https://gadm.org>).
